# An Effective and Safe Enkephalin Analog for Antinociception

**DOI:** 10.3390/pharmaceutics13070927

**Published:** 2021-06-22

**Authors:** K. K. DurgaRao Viswanadham, Roland Böttger, Lukas Hohenwarter, Anne Nguyen, Elham Rouhollahi, Alexander Smith, Yi-Hsuan Tsai, Yuan-Yu Chang, Christopher Llynard Ortiz, Lee-Wei Yang, Liliana Jimenez, Siyuan Li, Chan Hur, Shyh-Dar Li

**Affiliations:** 1 Faculty of Pharmaceutical Sciences, University of British Columbia, Vancouver, BC V6T 1Z3, Canada; kk.viswanadham@ubc.ca (K.K.D.V.); roland.boettger@web.de (R.B.); lukas.hohenwarter@alumni.ubc.ca (L.H.); anne.nguyen@alumni.ubc.ca (A.N.); elham.rouhollahi@ubc.com (E.R.); alexander.smith@ubc.ca (A.S.); liliana.jimenez@bcchr.ca (L.J.); siyuan.li@alumni.ubc.ca (S.L.); chan.hur@mail.mcgill.ca (C.H.); 2 Institute of Bioinformatics and Structural Biology, National Tsing-Hua University, Hsinchu 300, Taiwan; s108080541@m108.nthu.edu.tw (Y.-H.T.); cldortiz@gapp.nthu.edu.tw (C.L.O.); lwyang@life.nthu.edu.tw (L.-W.Y.); 3 Praexisio Taiwan Inc., Taipei 220, Taiwan; yuanyu.chang@praexisio.com.tw; 4 Chemical Biology and Molecular Biophysics Program, Taiwan International Graduate Program, Academia Sinica, Taipei 115, Taiwan; 5 Department of Chemistry, National Tsing-Hua University, Hsinchu 30013, Taiwan; 6 Physics Division, National Center for Theoretical Sciences, National Tsing Hua University, Hsinchu 300, Taiwan

**Keywords:** enkephalin, pain, analgesics, dependence, tolerance, respiratory suppression

## Abstract

Opioids account for 69,000 overdose deaths per annum worldwide and cause serious side effects. Safer analgesics are urgently needed. The endogenous opioid peptide Leu-Enkephalin (Leu-ENK) is ineffective when introduced peripherally due to poor stability and limited membrane permeability. We developed a focused library of Leu-ENK analogs containing small hydrophobic modifications. N-pivaloyl analog KK-103 showed the highest binding affinity to the delta opioid receptor (68% relative to Leu-ENK) and an extended plasma half-life of 37 h. In the murine hot-plate model, subcutaneous KK-103 showed 10-fold improved anticonception (142%MPE·h) compared to Leu-ENK (14%MPE·h). In the formalin model, KK-103 reduced the licking and biting time to ~50% relative to the vehicle group. KK-103 was shown to act through the opioid receptors in the central nervous system. In contrast to morphine, KK-103 was longer-lasting and did not induce breathing depression, physical dependence, and tolerance, showing potential as a safe and effective analgesic.

## 1. Introduction

Approximately 85% of people worldwide claim to have suffered from pain at some point in their lives [1]. Acute pain is a common symptom of diseases or injury. More than 50% of surgery or trauma patients experience severe to intolerable pain that, when poorly controlled, increases the risk of developing chronic pain, defined as pain persisting or recurring for more than 3 months [2]. Chronic pain affects nearly half of the world’s population and is one of the most common reasons for seeking medical advice, leading to estimated annual economic productivity losses of USD 560 billion in the United States alone [3]. Opioids are a class of pain killers used to treat severe pain by binding to and activating at least one of the opioid receptors (OR), including mu (MOR), delta (DOR), and kappa (KOR) opioid receptors. Extensive research has been conducted on developing new analgesics; however, up-to-date, MOR-activating alkaloids such as morphine are still among the most effective and widely used pain killers [4]. These alkaloid opioids are associated with significant side effects such as breathing depression, dependence, and tolerance [5]. Opioid abuse can lead to overdose death due to respiratory suppression. In 2018, 46,802 people died in the United States alone due to opioid overdose, representing approximately two-thirds of all drug overdose deaths [6]. A new analgesic that has a wide therapeutic window with fewer side effects is urgently needed.

Enkephalins (ENK, amino acid sequence: Tyr-Gly-Gly-Phe-Leu/Met) are small peptides produced endogenously and potent analgesics via OR activation. Leucine-ENK (Leu-ENK), in particular, is considered a promising alternative for pain treatment and potentially does not cause the side effects associated with alkaloid opioids such as morphine [7]. However, the direct use of ENKs for pain has been hampered by their low membrane permeability (low calculated logarithmic partition coefficient, clogP−0.85) and poor proteolytic stability leading to unfavorable pharmacokinetics (in vivo half-life ∼2 min) [5]. ENKs contain charges on the *N*- and *C*-termini at the physiological pH, and, thereby, are poorly membrane-permeable despite their hydrophobic side chains. Additionally, ENKs are rapidly degraded by exopeptidases in physiological fluids [8,9]. These shortcomings lead to low bioavailability and activity of ENKs when they are given peripherally. To overcome these limitations, several strategies to modify ENKs have been employed, but only with limited success [10]. Backbone modifications such as the insertion of a variety of isosteres [11,12,13,14,15,16] or unnatural d-amino acids [17] as well as macrocyclization [18,19] have been employed to improve the enzymatic stability; however, the in vivo results were disappointing due to the limited membrane permeability. Masking the termini with lipophilic moieties can eliminate the charges and increase the permeation through the biological membranes by passive transmembrane diffusion [20]. Additionally, since proteolytic degradation occurs mainly in the *N*- and *C*-termini [9], modification of these sites may also improve proteolytic stability. Previous studies mainly employed bulky terminal attachments such as fatty acids [21] and fluorenylmethyloxycarbonyl-based moieties [22]. Short *N*-alkyl modifications have been studied to analyze their impact on the receptor binding in vitro but were not investigated in vivo [23]. Moreover, in those prior studies, additional backbone modifications with d-amino acids [23,24,25], or a different linker between amino acids, such as squaric acid [12], cyclopropane [13], alkenes [14], triazoles [15], and thioethers [16], were also performed to enhance the proteolytic stability. The chemical structures of some of these previously reported ENK derivatives are summarized in Figure S1 (see Supporting Information).

Here, we synthesized novel Leu-ENK derivatives by introducing various short lipophilic groups to its *N*- and *C*-termini without altering the peptide backbone. We hypothesized that the small hydrophobic modifications would eliminate ionizable groups in the peptide and consequently improve the membrane permeation, while reducing the degradation by plasma proteases, leading to enhanced bioavailability. Since the modifications were minimal and the native core structure of Leu-ENK was reserved, the binding with the target receptors would be largely retained. As a result, the modifications would enhance the in vivo analgesic effect of Leu-ENK. This library of compounds was characterized and compared in vitro and in vivo, and a lead compound was identified. The efficacy and safety profiles of the lead compound were then compared with morphine in murine models.

## 2. Materials and Methods

### 2.1. Peptide Synthesis

All l-amino acids and solvents were analytical grade (>98%) and used without further purification as obtained from supplier VWR (Mississauga, ON, Canada). 1-Ethyl-3-(3-dimethylaminopropyl) carbodiimide hydrochloride (EDC·HCl), formic acid (FA), and silica gel were purchased from Sigma Aldrich (Oakville, ON, Canada). Peptide coupling reactions were performed under N_2_ atmosphere. THF was stored over KOH and freshly distilled prior to usage. The peptides were synthesized by conventional solution-phase methods using a racemization-free fragment condensation strategy. The *N*-Boc group was used for *N*-terminal protection, and the *C*-terminus was protected by methyl ester. Deprotection of the *N*-Boc group was performed using trifluoroacetic acid (TFA)/dichloromethane (DCM) at a 1:1 ratio (*v*/*v*) for 1 h, under N_2_ atmosphere. The intermediate TFA salts were used for subsequent reactions without further purification. Coupling was mediated by EDC·HCl and 1-hydroxybenzotriazole (HOBt). The detailed synthetic protocols for each intermediate and the final compounds are reported in the Supplementary Information. All intermediates and final compounds were characterized by ^1^H-NMR (400 MHz, Ascend NMR spectrometer, Bruker, Bremen, Germany) and reported as follows: chemical shifts in ppm downfield from tetramethylsilane as internal standard (δ scale), multiplicity (s = singlet, d = doublet, t = triplet, q = quadrate, m = multiplet and coupling constant (Hz)). All ^13^C-NMR spectra (200 MHz) are determined with complete proton decoupling and reported in ppm. NMR spectra of Leu-ENK analogs were provided in the supporting information (Figures S6–S23). Mass spectrometry was performed on an Electrospray-linear ion trap mass spectrometer (ESI-MS, QTrap 5500, potential 4500 V, AB Sciex, Concord, ON, Canada). The purity was characterized by analytical UPLC, and determined to be >95% (Supporting Information, Figure S24).

### 2.2. Peptide Stability

The in vitro stability of peptides was assessed according to a previously described protocol with modifications [26]. Peptides (3 g/L, 49 µL) dissolved in dimethylsulfamide (DMSO/TWEEN 80/normal saline (5/5/90, *v*/*v*/*v*) were diluted with pooled mouse dipotassium ethylenediaminetetraacetic acid containing pooled plasma (Innovative Research, Novi, MI, USA) or pooled human cerebrospinal fluid (CSF) (both BioIVT, Westbury, NY, USA) to a total volume of 500 µL. The final peptide concentration was kept constant at 315 µmol/L in all experiments. The mixture was incubated (37 °C, 750 rpm; Thermomixer, Eppendorf AG, Hamburg, Germany) and an aliquot (95 µL) was taken at 0, 5, 15, 60, and 300 min. For peptide quantification, the sample was mixed with aqueous acetonitrile (90%, *v*/*v*, 300 µL) containing FA, 0.1% *v*/*v* and incubated on ice for 30 min. After two centrifugation steps (21,100× *g*, 3 min, Sorvall Legend Micro 21R Centrifuge, Thermo Scientific, Waltham, MA, USA), the supernatant (300 µL) was collected, freeze-dried (FreeZone Triad Cascade Benchtop, Labcono, Kansas City, MO, USA), reconstituted in aqueous acetonitrile (4.5%, *v*/*v*) containing FA (0.1%, *v*/*v*), and stored at −20 °C. Samples were analyzed by ultra-performance liquid chromatography (UPLC).

### 2.3. UPLC

An ACQUITY UPLC H-Class chromatography system (Waters, Milford, MA, USA) coupled on-line to a photodiode array detector (absorbance recorded at 214 nm, Waters) and to the electrospray ionization source on a mass spectrometer (QDa detector, Waters) operated in the positive ion mode was used. Separation relied on a BEH-C18 column (inner diameter: 1.1 mm; length: 50 mm; particle size: 1.7 µm, Waters) at a flow rate of 0.1 mL/min using a linear aqueous acetonitrile gradient in the presence of FA (0.1%, *v*/*v*). Ionization was carried out at a source temperature of 450 °C using a cone voltage of 10 V and a capillary voltage of 1.5 kV. Mass spectra were typically acquired for an *m/z* range from 50 to 1250 at a sampling rate of 2 points per second. Quantities of the degraded peptides and resulting metabolites were estimated using peak areas of the UV signal at 214 nm relative to the initial peak areas (t = 0). Metabolites were identified using mass spectrometry by detecting the singly charged molecular ions.

### 2.4. Binding to DOR

The binding affinity of peptides to the DOR was performed by Eurofins Cerep SA (Celle-Lévescault, France) using a competitive radioligand displacement assay according to a previously described method [27]. Briefly, membrane preparations derived from COS-1 cells expressing DOR were incubated with [^3^H]DADLE (0.5 nmol/L), followed by treatment with a Leu-ENK analog at 6 nmol/L in tris(hydroxymethyl)aminomethane-HCl buffer (pH 7.4) for 1 h at the r.t. Radioligand displacement values were measured by scintillation and the binding affinity of the analog was expressed relative to native Leu-ENK.

### 2.5. Animals

Animal use and procedures were in accordance with the guidelines of The University of British Columbia animal care committee and the Canadian Council for Animal Care (CCAC, Protocol code: A17-0128, approval date: 30 August 2018). Experiments involved adult female CD-1 mice (25–30 g) housed under a 12 h light/dark cycle and had ad libitum access to rodent chow and water. Mice were allowed an acclimatization period of at least one week in the animal facility before experiments. Subcutaneous (s.c.) or intraperitoneal (i.p.) injections of drugs or vehicles were administered to animals at a volume of 10 mL/kg. In the formalin foot assay, each mouse was used once and euthanized immediately upon completion of the experiment due to irreversible tissue damage in the injected paw.

Throughout the eight-day duration of experiments investigating the development of physical dependence and tolerance, clinical health scores (CHS) of mice on a 5-point scale (0 normal and 5 highest severity) provided by the University of British Columbia Animal Care Committee were monitored. Scores were given in 5 categories (appearance (grooming, posture), behavior (gait, activity), local injection site skin appearance, neurological signs, and body weight). Based on the guidelines, mice with a score of 5 in any category or a cumulative score of >15, or body weight loss of >20% would be euthanized.

### 2.6. Hot-Plate Test

A commercially available hot-plate analgesia meter consisting of a round aluminum surface with (25 cm diameter) from Harvard Apparatus (Holliston, MA, USA) was used. A day before the experiment mice were acclimatized for 5 min in a transparent Plexiglas cylinder (height, 30 cm; diameter, 25 cm) on the switched-off hot-plate apparatus. Thereafter, the baseline latency of each animal was measured on the hot surface (52.0 ± 0.1 °C, 30 s cut-off time to prevent tissue damage) until a nociceptive response was induced. The animal was immediately removed from the hot surface after licking, lifting, or fluttering of the hind paws was observed. Latency responses after treatment were obtained in the same way. The %MPE for each animal was calculated using this equation: %MPE = (LR − BR)/(MR − BR)100%, where LR, MR, and BR represent measured, maximum (30 s), and baseline (~5 s) response time, respectively. The AUC for each animal was calculated from the %MPE time courses using this equation: AUC = (%MPE(Tp_1_) + %MPE(Tp_2_))·(Tp_2_ − Tp_1_)/2, where Tp represents the time interval between adjacent time points on the curve. Data were plotted as mean ± SEM.

### 2.7. Formalin Foot Assay

On the day of the experiment, mice were acclimatized to the experimental setup by putting them in darkened, non-transparent plexiglas cylinders (height, 20 cm; diameter, 9 cm) placed on a glass plane for 2 h. Mice received morphine (10 mg/kg, s.c.) or vehicle (s.c.) 5 min and KK-103 (13 mg/kg, s.c.) 150 min before injection of 20 µL of a 2.5% formalin solution into the intraplantar surface of the right hind paw using a 29G 0.5 mL insulin syringe (BD, Mississauga, ON, Canada). After successful injection, mice were immediately placed into cylinders and their behavior was monitored for 1 h. Video cameras (Lorex Technology, Markham, ON, Canada) underneath each animal were used to record the nociceptive responses defined as licking or biting at the formalin injected right paw. The accumulated licking and biting time of each animal was determined and recorded in 5 min bins. The total response time was plotted as early phase 1 from 0 to 5 min, and late phase 2 from 20 to 40 min after formalin injection.

### 2.8. Antagonist Co-Injection

Mice received an injection of KK-103 (13 mg/kg, s.c.) at 15 min post-injection of naloxone (Nlx, 3 mg/kg, i.p.), naloxone methiodide (Nlx-M, 4 mg/kg, i.p.), or vehicle. The thermal nociceptive response of mice was measured by the hot-plate method as described above.

### 2.9. Safety Test in Mice

The in vivo safety of KK-103 was studied by s.c. injecting a range of escalating doses of up to 650 mg/kg into mice. Mice were then monitored for mortality and clinical signs including respiratory activity, body weight, hydration, behavior (self-biting and circling), and pain assessment (Facial Grimace) to determine the maximum tolerated dose. The dose at 650 mg/kg was the maximum deliverable dose due to the limit of solubility in DMSO/TWEEN 80/H_2_O (5:5:95, *v*/*v*/*v*).

### 2.10. Respiratory Depression Assay

The respiratory rate was assessed in awake mice lightly restrained in a well-ventilated 50 mL falcon tube with a darkened tip. Prior to the drug treatment, each animal was habituated in the tube for 30 min. The falcon tube was secured onto a stand facing lengthwise towards a video camera. Animals (*n* = 6) were randomly assigned to receive an s.c. injection of either vehicle control, morphine (10 mg/kg), or KK-103 (13 mg/kg). Animals were recorded at selected time points for 10 min to measure the respiration rate.

### 2.11. Dependence Study

Mice (*n* = 6) received twice-daily injections of KK-103 (13 mg/kg), morphine (10 mg/kg), or vehicle at 10 a.m. and 3 p.m. for 8 consecutive days. Two hours after the last injection at 3 PM, mice received an i.p. injection of Nlx (4 mg/kg), after which they were immediately placed in a Plexiglas cylinder (height, 30 cm; diameter, 25 cm). The number of jumps induced by Nlx, defined as simultaneous removal of all four paws from the ground surface, were counted.

### 2.12. Tolerance Study

Mice (*n* = 6) were s.c. injected with KK-103 (13 mg/kg), morphine (10 mg/kg), or vehicle control twice daily at 10 a.m. and 3 p.m. for 7 consecutive days. The antinociceptive effect in mice was assessed on the hot-plate as previously described on days 1, 4, and 7. The analgesic activity (%MPE) was measured 15, 30, 60, 90, 120, 180, and 300 min post-injection, from which a time-effect curve was plotted and the AUC was calculated as described.

### 2.13. Statistical Analysis

Data presentation and statistical analysis are detailed in the figure legend. Statistical analysis was conducted using GraphPad Prism software (GraphPad Software, San Diego, CA, USA). A difference of *p* < 0.05 was considered to be statistically significant.

## 3. Results and Discussion

### 3.1. Design of Leu-ENK Analogs

**Figure 1:**
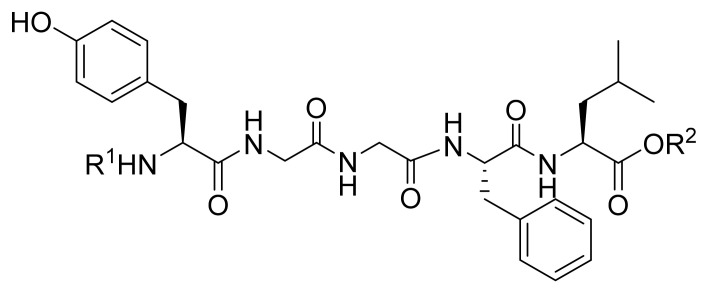
Chemical structure of Leu-ENK (sequence: YGGFL) and the sites of *N*-terminal (shown as R^1^) and *C*-terminal (shown as R^2^) modifications. The chemical structures of R^1^ and R^2^ within the general sequence R^1^HN-YGGFL-COOR^2^ are provided in Table 1.

As shown in Figure 1 and Table 1, we designed and synthesized a set of seven new analogs of Leu-ENK by attaching a small hydrophobic acyl moiety to the *N*-terminus. To further mask the charge on the carboxylic acid, we also attached a methyl ester to the *C*-terminus of Leu-ENK.

**Scheme 1:**
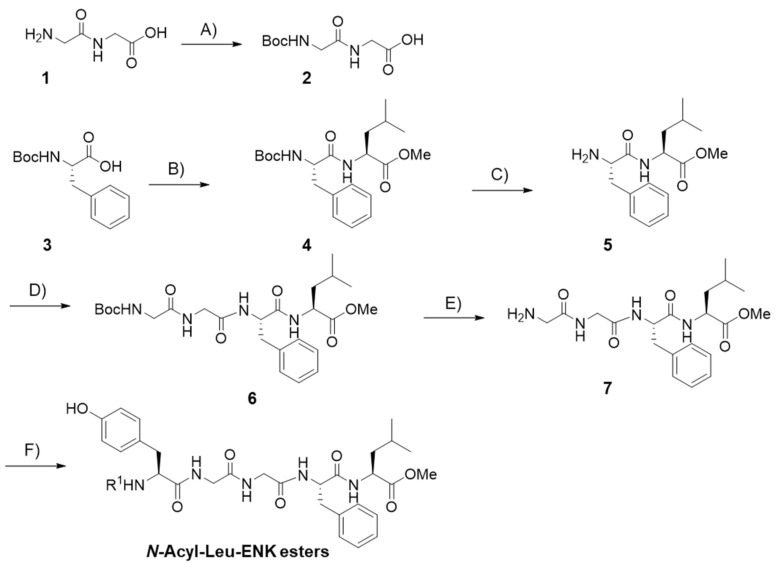
Solution-Phase Synthesis of Leu-ENK analogs. Reagents and conditions: (**A**) Boc anhydride, NaOH, (**B**) l-leucine methyl ester, EDC·HCl, HOBt, DMF, 0 °C, 10 min, triethylamine (TEA) then at room temperature (r.t.), overnight, then under N_2_ atmosphere; (**C**) TFA/DCM = 1:1 under N_2_ atmosphere, at r.t., 1 h; (**D**) B, Boc-glycyl-glycine **2**, intermediate **5**, EDC·HCl, HOBt, DMF, 0 °C, 10 min, then at r.t., overnight, under N_2_ atmosphere; (**E**) TFA/DCM = 1:1 under N_2_ atmosphere, r.t., 1 h; (**F**) *N*-acyl tyrosines (**8**–**14**, detailed structures can be found in the Supporting Information, Figure S2), EDC·HCl, HOBt, DMF, 0 °C, 10 min, TEA, then r.t., overnight, under N_2_ atmosphere.

Solution phase peptide synthesis was performed using the standard *N^α^-tert*-butyloxycarbonyl (Boc) strategy to yield Leu-ENK analogs (Scheme 1). The Boc protection of **1** gave corresponding Boc-Gly-Gly-OH (**2**). Boc-l-Phe-OH (**3**) was coupled with l-Leu-OMe to yield dipeptide Boc-l-Phe-l-Leu-OMe (**4**), followed by *N*-Boc-deprotection to give **5**. Dipeptides **2** and **5** were then subjected to the standard coupling reaction condition, which afforded tetrapeptide **6** in high yield (81.1%). Removal of the *N*-Boc protection of **6** was followed by coupling l-tyrosine with various *N*-terminal modifications (*N*-acyl tyrosine **8–14**, detailed structures can be found in the Supporting Information, Figure S2) to generate *N*-acyl-Leu-ENK esters. The chemical structures of these compounds are shown in Table 1. Leu-ENK analogs were purified by silica gel flash chromatography to give pure analogs as white powders in overall yields of 65–77%. The purity of the final analogs was ascertained by thin-layer chromatography (TLC), ^1^H-NMR, ^13^C-NMR. Final peptide purity was determined as >95% by analytical UPLC. 

We determined the lipophilicity and metabolic stability of the analogs and compared them with native Leu-ENK. First, the lipophilicity (clogP) of the analogs was calculated by ChemBioDraw. As shown in Table 1, the modifications increased the clogP value from −0.85 to 1.34–3.70, suggesting improved membrane permeability. Proteolytic degradation is a major hurdle for many peptides to exert their in vivo efficacy [29]. We tested the stability of Leu-ENK in CD-1 mouse plasma and determined that only 23 ± 3% of Leu-ENK remained intact after 1 h incubation.

Leu-ENK (one letter code: H_2_N-YGGFL-COOH) was rapidly degraded to H_2_N-GGFL-COOH and H_2_N-FL-COOH (Supporting Information, Figure S3), indicating rapid degradation by aminopeptidases and dipeptidyl aminopeptidases from the *N*-terminus. The result is consistent with the previous studies that investigated the hydrolysis sequence of Leu-ENK in plasma [8,9]. Roscetti et al., 1985 [8] showed that initially Tyr is cleaved followed by simultaneous cleavage of the Gly–Phe and Gly–Gly bonds to release different C-terminally truncated sequences that are further degraded into their amino acid building blocks. Hence, the onset of Leu-ENK metabolization mainly proceeds by aminopeptidases from the N-terminus of the peptide [9].

**Figure 2:**
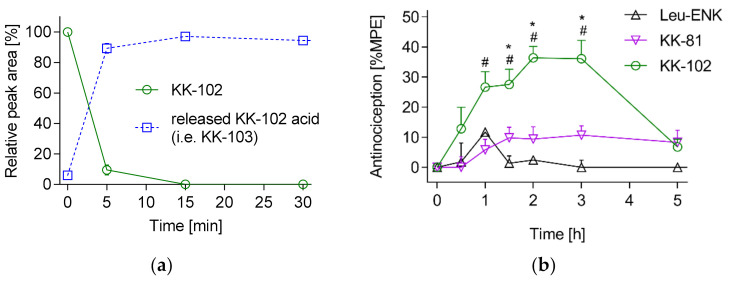
Stability of KK-102 containing a *C*-terminal ester and release of the corresponding acid KK-103 in female CD-1 mouse plasma (**a**) and antinociceptive activity in the hot-plate model on female CD-1 mice comparing the percent %MPE of three representative Leu-ENK analogs (13 mg/kg, s.c. injection, panel **b**). Data = mean ± SEM (a: *n* = 3, b: *n* = 6). Statistical comparisons were performed using a two-way ANOVA with multiple comparisons and Tukey post hoc test. * *p* < 0.001: KK-102 compared to Leu-ENK, # *p* < 0.001: KK-102 compared to KK-81.

We then further examined the plasma stability of the analogs and discovered that the *C*-terminal methyl ester was rapidly hydrolyzed within minutes, and the released acid (i.e., Leu-ENK with an *N*-terminal modification only) was relatively stable in the plasma. This was exemplarily shown for KK-102, which was degraded with a half-life of 2.5 min releasing the corresponding KK-102 acid, which was later named KK-103 (Figure 2a). The released KK-103 was then not further truncated in plasma within the experimental time frame. After 1 h incubation in plasma, 94 ± 5% of KK-103 converted from KK-102 remained. This rapid ester hydrolysis releasing a relatively stable acid was observed with all the Leu-ENK analogs. Among all analogs, 51–88% of the original peptide amount was recovered as the corresponding acid after 1 h incubation in plasma, and KK-102 displayed the highest plasma stability among the tested analogs (Table 1). Interestingly, we did not detect the same *C*-terminal metabolites (H_2_N-FL-COOH and H_2_N-GGFL-COOH) as for native Leu-ENK. Instead, we identified the *N*-terminal metabolites R^1^HN-Y-COOH, R^1^HN-YGG-COOH, and R^1^HN-YGGF-COOH containing the corresponding *N*-terminal modifications (Supporting Information, Figure S4). Hence, the data suggest that the *N*-terminal modifications efficiently reduced *N*-terminal truncation by aminopeptidases, which aligns with previous studies on *N*-acetyl-Enkephalin. Jayawardene et al. 1999 [28] showed that *N*-terminal acylation with a variety of modifications including Acetyl prevents the peptide truncation by aminopeptidase M leading to high recovery rates of the peptides after incubation in plasma for at least 8 h. On the other hand, *C*-terminal truncation taking place at a slower rate was observed for these analogs after one hour, which was not detected for Leu-ENK, as it was metabolized at the *N*-terminus before that.

To study whether the introduced modifications would impair the binding with the receptor, we screened the binding to human DOR by a radioligand competition assay using [^3^H]-d-Ala2-d-Leu5-enkephalin (DADLE) at the concentration of its K_D_ value of 0.6 nM. We selected DOR for the binding assay because Leu-ENK displays the highest affinity for DOR among all OR subtypes. As shown in Table 1, all modifications resulted in a reduced relative binding affinity compared to native Leu-ENK. The seven analogs showed 29–70% relative DOR binding affinity compared to Leu-ENK, with KK-102 showing the strongest binding (70% relative to Leu-ENK). Finally, we studied the antinociceptive activity of those analogs in a hot-plate test in mice after s.c. administration at a dose of 20 µmol/kg (~13 mg/kg). All modified analogs displayed increased antinociception compared to native Leu-ENK with a 2.6- to 8-fold increase of the AUC. (Table 1) Figure 2b depicts two representative analogs KK-81 and KK-102 and Leu-ENK showing AUCs of 37, 112, and 14%MPE·h, respectively. Again, KK-102 showed the highest activity among all analogs being 8-fold higher than that of Leu-ENK. Interestingly, while KK-102 was shown to rapidly convert to KK-103 in plasma and their overall antinociceptive effect (i.e., AUC) was comparable (Figure 2b and Table 1), their efficacy profiles were different. The effect of KK-103 persisted at 5 h, but the KK-102 effect dropped significantly at 5 h, the reason for which is to be investigated.

**Figure 3:**
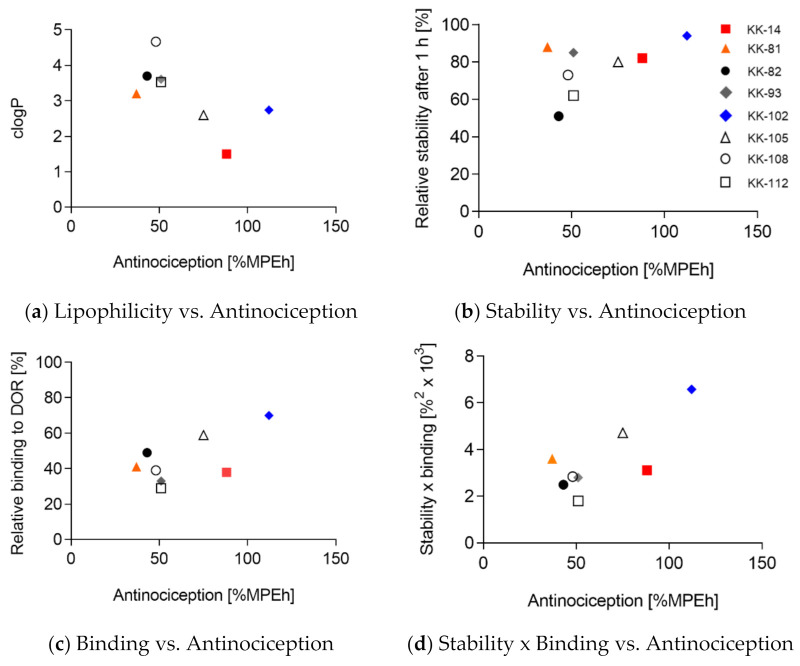
Pearson correlation between the AUC antinociception and (**a**) lipophilicity (clogP), (**b**) plasma stability, (**c**) DOR binding and (**d**) plasma stability × DOR binding of the Leu-ENK analogs.

We correlated the in vitro characteristics (clogP, plasma stability, relative binding affinity, Figure 3a–c) of the eight analogs with their in vivo efficacy to examine which pharmaceutical property was critical for improving Leu-ENK.

None of those parameters alone showed a statistically significant correlation (Table 2), and surprisingly clogP even displayed a trend of negative correlation. The combination of stability and binding correlated with antinociception with an R^2^ of 0.59 and *p* = 0.03 (Figure 3d, Table 2). This analysis demonstrates that the increased activity of the analogs cannot be explained by a single property, but rather a combination of stability and receptor binding.

Those improvements over native Leu-ENK are attributed to the introduced *N*-terminal modifications, which are small compared to previously reported bulky *N*-acyl-Leu-ENK analogs that showed activity in vivo [21,22]. Additionally, it has been shown that adding amino acids to the *N*-terminus of Leu-ENK does not result in loss of activity [30]. Summers et al. investigated the binding affinity of *N*-alkylated Met-ENK for mouse DOR and found that *N*-hexyl derivatives displayed the strongest binding and that analogs with a shorter or longer alkyl chain showed decreased binding [23]. Similarly, among the Leu-ENK analogs investigated in this study, *N*-pivaloyl-Leu-ENK (KK-102) exhibited the strongest DOR binding (70% relative to Leu-ENK), while analogs with a smaller (*N*-acetyl: KK-14) or larger *N*-alkylated modification (*N*-pentenoyl: KK-105; *N*-hexanoyl: KK-93; *N*-octanoyl: KK108) displayed decreased DOR binding (30–60% relative to Leu-ENK). The bulkier *N*-phenylacetyl (KK-81), *N*-tolylacetyl (KK-82), and *N*-cyclohexanoyl (KK-112) also showed reduced binding affinity compared to KK-102. Our results agree with the previous report and suggest that there is an optimal size of the *N*-terminal modification to preserve binding affinity with DOR [23].

Proteolytic degradation of peptides is mediated by binding to the active site of a protease [31], and, therefore, introducing steric hindrance around the cleavage site can reduce recognition by the protease and increase the proteolytic stability [32]. It is generally agreed that modifications at approximately four amino acids from the cleaved bond of the substrate can significantly increase the proteolytic stability [33,34], Indeed, we showed that none of the *N*-acyl analogs displayed degradation at the *N*-terminus, and this is likely attributed to the steric hindrance. The results are in line with previous reports utilizing a range of *N*-terminal modifications such as *N*-phosphorylation [35], *N*-acetylation [28], 4-imidazolidinone prodrugs [36], and squalene conjugates [37]. Interestingly, we found that even a small difference in the modifications such as a single methyl group distinguishing the *N*-phenylacetyl (KK-81) and *N*-tolylacetyl (KK-82) analogs resulted in significantly different plasma stability. While KK-81 was more stable than KK-82 and exclusively truncated at the *C*-terminal Leu, KK-82 exhibited further degradation sites between the Tyr-Gly and Gly-Phe bonds (Supporting Information, Figure S4). Our results indicate that modifications more than four amino acids apart from the cleaved site also have an impact on the protease cleavage.

As the ester analogs were rapidly converted into the corresponding acids, which were relatively stable in plasma and exhibited improved activity in vivo, we hypothesized that the *C*-terminal methyl ester modification was not essential for activity. We, therefore, synthesized the acid version of our lead compound KK-102, which is the corresponding acid KK-103, and evaluated its pharmaceutical properties. KK-103 was synthesized by hydrolysis of the *C*-terminal methyl ester of KK-102 using LiOH in tetrahydrofuran (THF)/H_2_O [38,39]. KK-103 showed an almost identical relative DOR binding (68 ± 2% vs. 70 ± 1%) compared to its parent compound KK-102, both of which exerted a receptor binding that was most comparable to Leu-ENK (Table 1). This experimental data is supported by our in silico docking results that suggest a high similarity between Leu-ENK and KK-103 with respect to binding to DOR (Supporting Information Table S1, Figure S5). As a result, the in vivo efficacy of KK-103 was comparable to KK-102 (142 ± 15%MPE·h vs. 112 ± 24%MPE·h).

**Figure 4:**
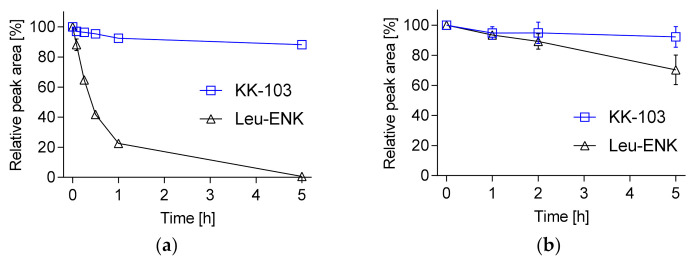
In vitro stability of KK-103 compared to native Leu-ENK in (**a**) plasma and (**b**) CSF. Data = mean ± SEM, *n* = 3. SEM bars are narrow and are within the data symbol.

We then focused on investigating the analgesic mechanism, efficacy, and safety of KK-103 in mice. First, we compared the stability of KK-103 and Leu-ENK pooled plasma in vitro. As shown in Figure 4a, Leu-ENK was rapidly degraded in plasma and the concentration was undetectable after 5 h of incubation with a plasma half-life of 25 min. On the other hand, more than 90% of intact KK-103 remained in the plasma after 5 h of incubation, displaying an extrapolated half-life of 37 h, a ~90-fold increase over Leu-ENK. We also compared their stability in the CSF and determined that more than 95% KK-103 and 80% Leu-ENK were recovered after 5 h of incubation, indicating both compounds were stable in the CSF (Figure 4b). This is in line with previous investigations demonstrating high stability of ENKs incubated in CSF for at least 5 h at 37 °C [40], while the half-life decreased to 4 min in the presence of brain tissue [41]. The peptidases responsible for enkephalin catabolism such as aminopeptidases, dipeptidylaminopeptidases, angiotensin-converting enzyme, and enkephalinase are cell membrane-bound and merely present in the soluble fraction of CSF [42]. On the other hand, the plasma contains soluble versions of these enzymes accounting for the low stability of ENKs in this matrix [8]. Overall, the increased stability of KK-103 in plasma suggests that the analog exerted its analgesic activity by itself rather than through conversion back to Leu-ENK in vivo.

### 3.2. Analgesic Effect of KK-103 Compared to Morphine

**Figure 5:**
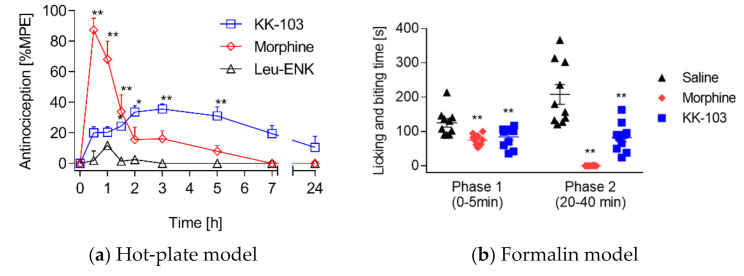

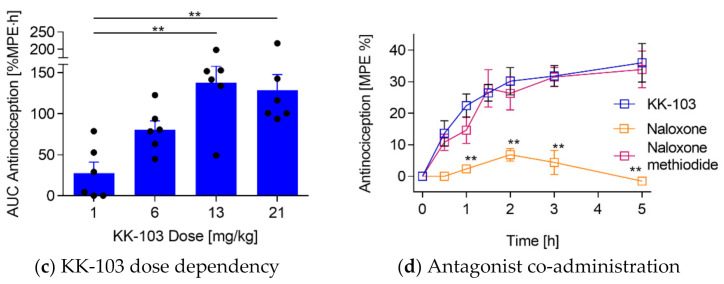
Antinociceptive activity in female CD-1 mice with s.c. injection of drugs. (**a**) Hot-plate model comparing the %MPE of Leu-ENK (13 mg/kg), KK-103 (13 mg/kg) and morphine (10 mg/kg), ** *p* < 0.001, * *p* < 0.02 compared to Leu-ENK. (**b**) Formalin injection test comparing KK-103 (13 mg/kg), morphine (10 mg/kg) and vehicle with responses representing the total time of mice spent licking and biting in phase 1 (0–5 min) and phase 2 (20–40 min) of the nociceptive response to formalin injection. ** *p* < 0.001 compared to vehicle. (**c**) Dose-effect relationship of KK-103 in the hot-plate model with overall antinociception represented as the AUC of the %MPE plot, ** *p* < 0.001, n.s denotes not significant. (**d**) Hot-plate model comparing the activity of co-administrated antagonist Nlx (i.p. 3 mg/kg), or Nlx-M (i.p. 4 mg/kg) with K-103 (13 mg/kg) + vehicle, ** *p* < 0.05. Data = mean ± SEM (*n* = 6) for (**a**–**c**); Data = mean ± 95% confidence interval (*n* = 10) for (**b**). Statistical comparisons were performed using a two-way ANOVA with multiple comparisons and Tukey post hoc test (**a**,**d**), one-way ANOVA with Tukey post hoc test (**b**,**c**).

In this report, we mainly employed the hot-plate test to quantify analgesic activity in mice, as it is the easiest, most robust, and commonly used method. The hot-plate method applies thermal stimulus (~52 °C) to the paws of mice and induces nociceptive responses that can be recorded, including paw licking and jumping. A treatment that increases nociceptive response time in mice is regarded as effective [43]. Using this assay, we compared the analgesic effects of Leu-ENK, KK-103, and morphine, the most commonly used and characterized opioid analgesic [44]. As shown in Figure 5a, Leu-ENK administered s.c showed little analgesic effect, which peaked at 1 h with antinociception of ~10%MPE and then declined to the baseline in 1.5 h. Morphine administered s.c. at 10 mg/kg showed a rapid onset and reached its maximum at 87%MPE within 15 min, but decreased to ~10%MPE in 2 h. The data are in line with previous reports such as Pacifici et al. reporting a peak effect in C57BL6 mice at 20 min [45]. In contrast, KK-103 given s.c. at 13 mg/kg showed a slower onset but longer-lasting antinociceptive effect: the efficacy peaked at 2 h post-injection (30–40%MPE), followed by a plateau until 5 h and then slowly declined to baseline in 24 h. The AUCs were 14 ± 6, 142 ± 15, and 138 ± 12%MPE·h for Leu-ENK, KK-103, morphine, respectively. While the AUC of antinociceptive efficacy of morphine and KK-103 were comparable, these compounds demonstrated very different efficacy profiles. In contrast to the immediate strong effect of morphine, KK-103 showed a weaker, but longer-lasting effect. The mechanism of the prolonged efficacy of KK-103 is yet to be studied.

In addition to the hot-plate test, the formalin injection model was employed to compare the analgesic activity of KK-103 with morphine. This test uses a chemical noxious stimulus, which produces a longer-lasting and progressing nociception compared to thermal tests, making it more clinically relevant. Due to its simplicity and the reproducibility of the pain-related behaviors, the formalin injection test is commonly used for the preclinical screening of analgesics. The measured parameter is the cumulative time that rodents spend licking or biting the formalin-injected paw in response to the stimulus [46]. The behavior of animals after intraplantar injection of formalin is biphasic, resulting from direct stimulation of nociceptors in the first phase and inflammatory response in the second phase [47]. While nonsteroidal anti-inflammatory drugs only suppress the second phase, opioid analgesics seem to have an antinociceptive effect in both phases, which makes this test well-suited for substances acting on ORs [48]. As shown in Figure 5b, in phase 1 from 0 to 5 min, the paw licking and biting time was reduced by 30% in morphine, and 25% in KK-103 treated mice compared to the control group. In phase 2, KK-103 significantly reduced the licking and biting time to ~50% of the mean response of the vehicle group, while morphine completely diminished the nociceptive response in all mice. While both morphine and KK-103 were effective in both phases, the phase 2 effect appeared to be more significant than that in phase 1 for both drugs. Overall, these results clearly showed the effectiveness of KK-103 in this inflammation-related pain model.

Next, we examined the dose-dependent efficacy of KK-103. KK-103 was applied s.c. at four different doses ranging from 1 mg/kg to 21 mg/kg, and the analgesic effect was measured by the hot-plate test and expressed as the AUC as described above. As shown in Figure 5c, the effect of KK-103 increased with the dose and peaked at 13 mg/kg, and an increase of dose to 21 mg/kg did not further enhance antinociception compared to 13 mg/kg. The AUCs for KK-103 at 1, 6, 13, and 21 mg/kg were 28 ± 13, 81 ± 11, 138 ± 20, and 129 ± 19%MPE·h, respectively.

To examine the mechanism of KK-103, we co-injected KK-103 with Nlx, a highly membrane-permeable pan-OR antagonist, or with Nlx-M, a membrane-impermeable and peripherally restricted OR antagonist, and measured the analgesic activity using the hot-plate method. This approach has been widely used for studies of the mechanism of action for opioids [49]. Figure 5d shows that co-injection with Nlx significantly reduced KK-103′s analgesic activity, while Nlx-M showed no effect on the efficacy of KK-103. The results suggest that KK-103 exerted its analgesic activity through the ORs in the central nervous system (CNS). Although KK-103 displayed a good affinity for DOR (Table 1), our current data set could not conclude that the antinociceptive efficacy of KK-103 was entirely attributed to the DOR activation. Additional investigations of KK-103 binding and activation on subtype ORs, including MOR, DOR, and KOR, are needed to obtain a comprehensive understanding of KK-103′s mechanism of action and to complement our efficacy and safety data in vivo.

It is noted that the antinociceptive activity of OR agonists is sex-dependent, producing a more decreased effect in female than in male rodents [50]. While this study focused on female rodents for the purpose of compound screening, the sex difference for KK-103 activity will be addressed in future studies. 

### 3.3. Safety Profiles of KK-103 Compared to Morphine

The safety of KK-103 was evaluated using various tests. First, we gradually increased the dose of KK-103 and monitored the mice using clinical scores, which examined respiration, body weight, hydration, behavior (self-biting and circling), and pain. Due to the solubility limit of KK-103, the maximum deliverable dose was 650 mg/kg. Mice treated at this dose did not show any signs of abnormal behaviors (e.g., hyperactivity) or an increase in the clinical scores. Hence, KK-103 was safe even at its maximum deliverable dose of 650 mg/kg, which is 50-fold higher than the effective dose (13 mg/kg). On the other hand, the maximum tolerated dose of morphine was 130 mg/kg [51], which was only 13-fold higher compared to its therapeutic dose (10 mg/kg). Therefore, the therapeutic window of KK-103 was at least 4-fold higher than that of morphine.

**Figure 6:**
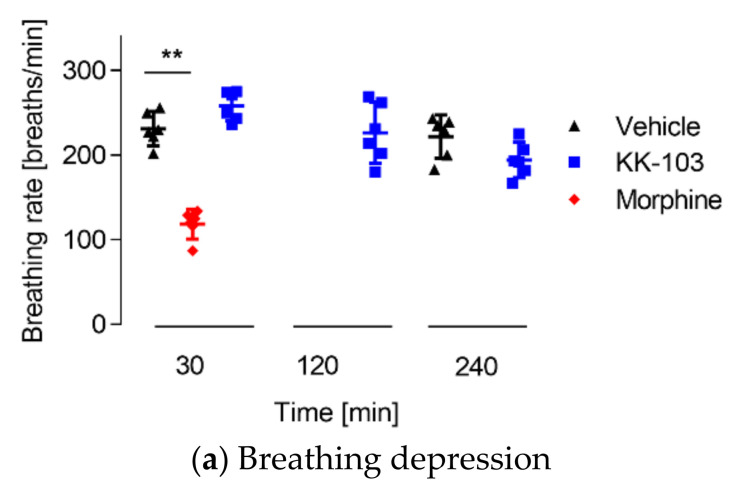

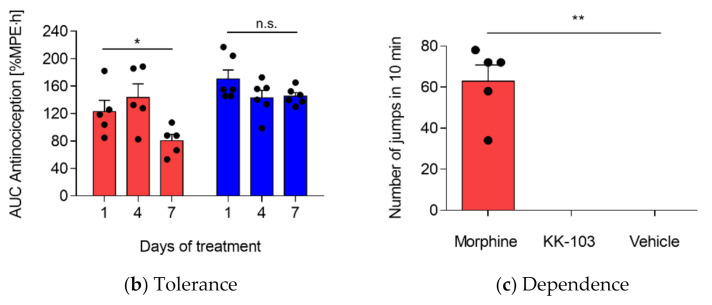
Safety profiles of KK-103 (s.c., 13 mg/kg) compared to morphine (s.c., 10 mg/kg) in female CD-1 mice. (**a**) Breathing effect of KK-103 and morphine were compared to vehicle at different time points after drug administration. ** *p* < 0.001 compared to vehicle. (**b**) Antinociceptive tolerance measured by the hot-plate test. Mice received twice-daily treatments for 7 consecutive days. The analgesic activity was measured on days 1, 4, and 7, * *p* < 0.01 (morphine day 1 vs. day 7), n.s. denotes not significant (KK-103, day 1 vs. day 7). (**c**) Physical dependence in mice after receiving twice-daily treatments for 8 days. Two hours post the final dose, mice received Nlx (i.p., 4 mg/kg) and the number of jumps within the first 10 min were measured. ** *p* < 0.001 compared to vehicle Data = mean ± 95% confidence interval (*n* = 10) for panel (**a**) and data = mean ± SEM (*n* = 6) for panels (**b**,**c**). Statistical comparisons were performed using one-way ANOVA with Tukey post hoc test for the 30 min time point and two-tailed unpaired t-test for 120 and 240 min (**a**), one-way ANOVA with Tukey post hoc test (**b**,**c**).

We also compared specific side effects between morphine and KK-103, including respiratory suppression, dependence, and tolerance. Breathing suppression is the leading cause of death after acute morphine poisoning and significantly limits the dose that can be safely administrated [52]. Therefore, breathing rate decrease is considered the most concerning and dangerous side effect of opioids [52]. Animals (*n* = 6) were randomly assigned to receive either vehicle, KK-103 (13 mg/kg), or morphine (10 mg/kg) via s.c. injection and their breathing rates were measured. As shown in Figure 6a, morphine significantly decreased the respiratory rate by 56% compared with the vehicle control at 30 min post-injection, which is in line with previous reports. Hill et al. [53,54] reported 1.7-fold decreased breathing rates at an equivalent dose of morphine 30 min after administration in mice. In contrast, the respiration rate of KK-103-treated mice was comparable to that of mice treated with vehicle at any time point between 30 and 240 min.

Another adverse effect that complicates the use of opioids, especially after repeated use, is the development of physical dependence, which is characterized by the occurrence of withdrawal symptoms after opioid cessation [55]. Repeated administration of morphine and other opioids leads to the adaptation of certain neuronal processes and signaling pathways, which become over-activated when opioids are discontinued.

This results in withdrawal symptoms that include muscle cramps and anxiety, which motivate the continuation of opioids to alleviate these side effects. The withdrawal symptoms that are only associated with physical discomforts are known as physical dependence [55]. We compared the development of physical dependence of KK-103 with morphine. Female CD-1 mice were injected twice daily with morphine (10 mg/kg), KK-103 (13 mg/kg), or vehicle for eight days. After the last treatment, withdrawal, which in mice is associated with jumping behavior, was induced by an i.p. dose of Nlx at 4 mg/kg [56]. The number of jumps of each mouse in the first 10 min post-Nlx injection was measured. As shown in Figure 6b, repeated administrations of morphine-induced significant withdrawal syndromes in mice following Nlx challenge, indicating opioid dependence.

This is in line with previous reports assessing the dependence of morphine using the same method [57]. The group treated with KK-103, on the other hand, did not show any signs of physical dependence, i.e., not a single jump was observed.

Tolerance is another common side effect of chronic opioid use and involves multiple mechanisms that primarily result in a gradual loss of analgesic potency and efficacy. Consequentially, a higher dose will be needed to maintain the same level of analgesia, and this dose increase often leads to increased side effects [58,59,60]. Therefore, we investigated whether mice developed tolerance to KK-103 and morphine after chronic use. In the present study, the development of antinociceptive tolerance was assessed following s.c. administrations of morphine (10 mg/kg) or KK-103 (13 mg/kg) twice daily for 7 consecutive days. As shown in Figure 6c, the overall antinociceptive activity of morphine on day 4 remained unchanged compared to day 1 (123 vs. 143%MPE·h, respectively) but was significantly decreased by 35% on day 7 (80%MPE·h), indicating the development of tolerance on day 7. This is consistent with previous studies demonstrating the establishment of tolerance after morphine use in mice [61,62]. Mansouri et al. [61] reported a residual effect of morphine of approximately 30% at day six of the treatment in an equivalent animal model. In contrast, KK-103 remained equally efficacious after seven days of treatments (170 vs. 145%MPE·h, day 1 and day 7, respectively), indicating no development of tolerance. CHS remained 0 for KK-103 treated mice during the multidose tolerance and physical dependence tests.

The data suggest that KK-103 was a safer analgesic than morphine. The improved safety of KK-103 might be explained by its high structural similarity to the endogenously produced Leu-ENK. Indeed, various compounds harnessing the endogenous ENK system have been demonstrated to exhibit improved safety without inducing typical side effects caused by MOR agonists, including respiratory depression, tolerance, physical dependence, and constipation [63,64,65,66,67,68,69,70,71].

Several ENK analogs have been synthesized and shown to produce analgesia with reduced or little side effects on constipation, dependence, tolerance, and locomotor activity [63,68,69,72]. Godfrey et al. [71] developed a chitosan-based nanoparticle formulation for intranasal delivery of native ENK and showed strong antinociception without inducing tolerance or hyperactivity after repeated administrations. Similarly, dual enkephalinase inhibitors, which increase the extracellular level of ENKs were shown to produce antinociception without respiratory disturbance, constipation, analgesic tolerance, addiction, or dependence in several animal models [64,65,66,67,70,73,74,75].

## 4. Conclusions

In this study, we demonstrated that pivaloyl conjugation to the *N*-terminus of Leu-ENK significantly increased the plasma stability by 90-fold, while largely maintaining the binding affinity for the DOR, leading to a 10-fold increased antinociceptive activity in mice. KK-103 exerted its antinociceptive activity via ORs in the CNS with dose-dependent efficacy. While its AUC over the time course of activity is comparable to morphine, KK-103 was slower acting but longer lasting. KK-103 was effective in both thermal and chemical noxious stimulus models. Compared to morphine, KK-103 exhibited a >4-fold increased therapeutic window and did not induce breathing suppression, physical dependence, and tolerance. Our data indicate KK-103 was an effective and safe analgesic in mice models.

## Figures and Tables

**Table 1:** In vitro characteristics and in vivo antinociceptive activity of Leu-ENK analogs (general sequence: R^1^HN-YGGFL-COOR^2^).

Compound Code	*N*-Terminal Modification R ^1,a^	*C*-Terminal Modification R ^2,a^	clogP ^b^	Recovered Peptide after 1 h in Mouse Plasma [%]	Relative Binding to DOR Compared to Leu-ENK [%] ^c^	Antinociception (AUC_0-5h_, %MPE·h) ^d^
Leu-ENK	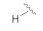	H	−0.85	23 ± 2	100 ± 16	14 ± 6
KK-14 ^e^	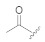	CH_3_	1.50	82 ± 5	38 ± 4	89 ± 28
KK-81	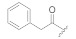	CH_3_	3.20	88 ± 7	41 ± 25	37 ± 10
KK-82	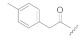	CH_3_	3.70	51 ± 5	49 ± 20	43 ± 17
KK-93	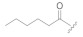	CH_3_	3.61	85 ± 7	33 ± 11	51 ± 13
KK-102	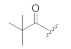	CH_3_	2.74	94 ± 3	70 ± 11	112 ± 24
KK-105	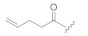	CH_3_	2.60	80 ± 7	59 ± 22	75 ± 22
KK-108	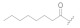	CH_3_	4.67	73 ± 4	39 ± 27	48 ± 15
KK-112	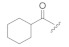	CH_3_	3.53	62 ± 4	29 ± 21	51 ± 10
KK-103 ^f^	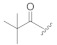	H	2.61	93 ± 1	68 ± 2	142 ± 15

^a^ Full structure is shown in Figure 1. ^b^ Calculated using ChemBioDraw 12.0. ^c^ The binding to human DOR measured as relative scintillation compared to Leu-ENK at 6 nM in a cell-based competitive radioligand displacement assay. ^d^ The antinociceptive activity in female CD-1 mice is expressed as the area under the efficiency curve (AUC) of the antinociceptive activity plot (percent maximal possible effect, %MPE vs. time) using a hot-plate test after s.c. injection of compounds. %MPE = (LR-BR)/(MR-BR)100%, where LR, MR, and BR represent measured, maximum (30 s), and baseline (~5 s) response time, respectively. Data = mean ± SEM, *n* = 6. ^e^ as previously synthesized in reference [28]. ^f^ Peptide containing a free *C*-terminal acid, released from the corresponding ester precursor.

**Table 2:** Summary of the statistical values of the Pearson correlation performed on the scatter plots in Figure 3. n.s. denotes not significant, * *p* < 0.05.

	R^2^	*p* (Two-Tailed)	*p* Value Summary
Lipophilicity vs. Antinociception	0.4376	0.07	n.s.
Stability vs. Antinociception	0.2777	0.18	n.s.
Binding vs. Antinociception	0.4372	0.07	n.s.
Stability × Binding vs. Antinociception	0.5909	0.03	*

## Data Availability

The data presented in this study are available on request from the corresponding author.

## Supplementary Materials

The following are available online at https://www.mdpi.com/article/10.3390/pharmaceutics13070927/s1, Figure S1: Structure of previously reported Leu-ENK derivatives, Figure S2: Structures of N-acyl tyrosine, Figure S3: The stability of Leu-ENK in mouse plasma, Figure S4: The stability of Leu-ENK analogs in mouse plasma, Additional experimental procedures, Table S1: Binding Energies of Leu-ENK and KK-103 to DOR, Figure S5: Molecular binding data of KK-103 and Leu-ENK, Figure S6–S23: Copies of 1H and 13C-NMR spectrum of Leu-ENK analog, Figure S24: UPLC purity data for KK-103, and, supporting results and discussion: molecular binding of peptides to DOR, and solution-phase synthesis of Leu-ENK analogs.
