# Supplementary material for: An Effective and Safe Enkephalin Analog for Antinociception
Source: Pharmaceutics. 2021 Jun 22;13(7):927. doi: 10.3390/pharmaceutics13070927 (PMC8308721; doi:10.3390/pharmaceutics13070927)
Supplement: Supplementary file 1 [file pharmaceutics-13-00927-s001.zip › pharmaceutics-1246226-SI.pdf]

pharmaceutics

MDPI

Supplementary Materials: An Effective and Safe Enkephalin Analog for Antinociception

KK DurgaRao Viswanadham, Roland Böttger, Lukas Hohenwarter, Anne Nguyen, Elham Rouhollahi, Alexander Smith, Yi-Hsuan Tsai, Yuan-Yu Chang, Christopher Llynard Ortiz, Lee-Wei Yang, Liliana Jimenez, Siyuan Li, Chan Hur and Shyh-Dar Li

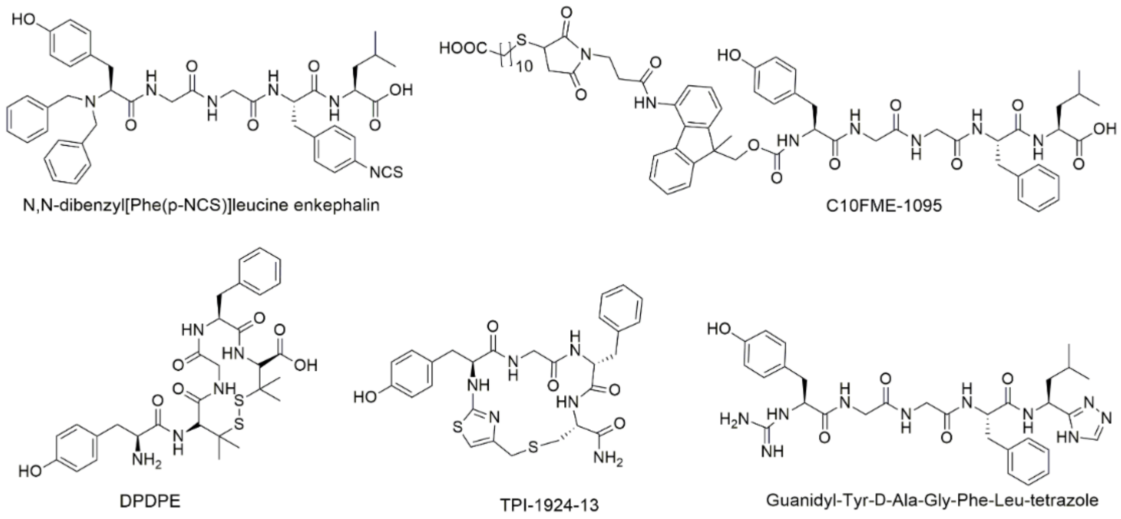

Figure S1. Structures of previously reported Leu-ENK derivatives; *N*,*N*-dibenzyl[Phe(p-NCS)]leucine enkephaline[1]; C10FME-1095[2]; DPDPE[3], TPI-1924-13[4] and Guanidyl-Tyr-D-Ala-Gly-Phe-Leu-tetrazole[5].

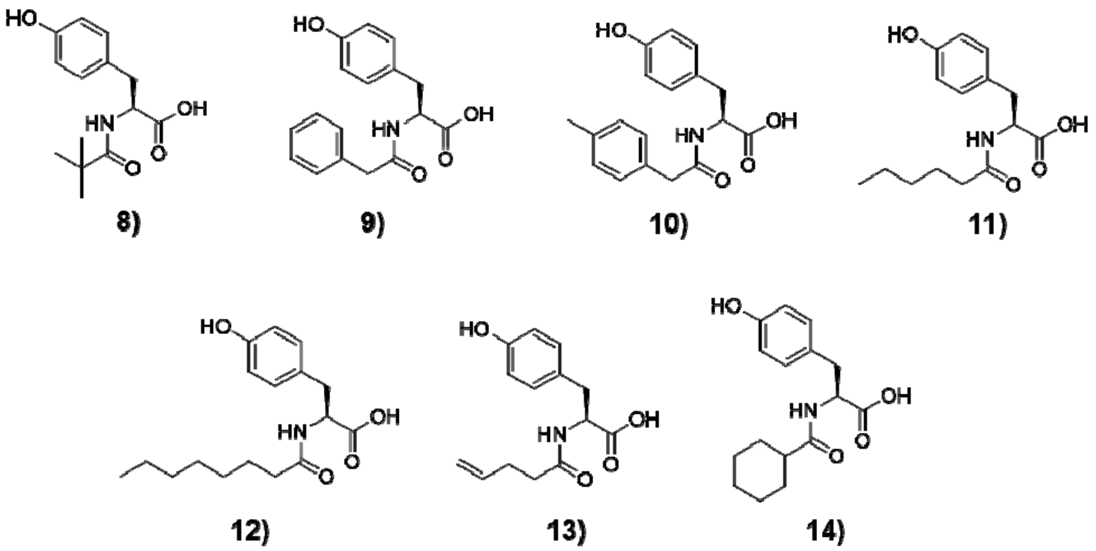

Figure S2. Structures of *N*-acyl-tyrosine intermediates used in this study.

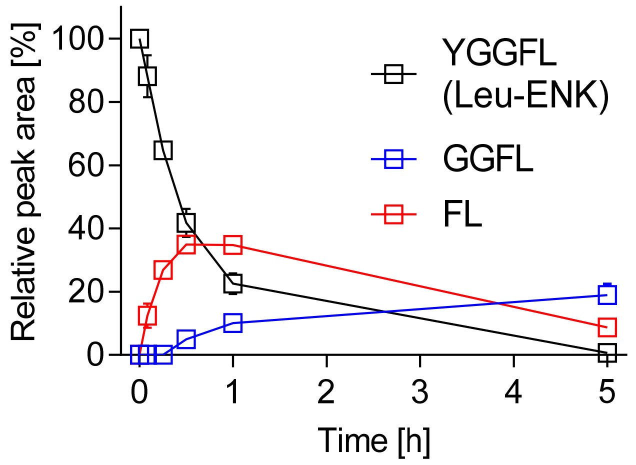

Figure S3. Stability of Leu-ENK (black) in mouse plasma and release of the two major metabolites Leu-ENK (2-5, sequence: GGFL, red) and Leu-ENK (4-5, sequence: FL, blue). Samples were separated by UPLC and peptides were detected by their absorbance at 214 nm. Quantification relied on the peak areas relative to the initial amount of peptide (t = 0 min). Values represent mean ± SEM (*n* = 3).

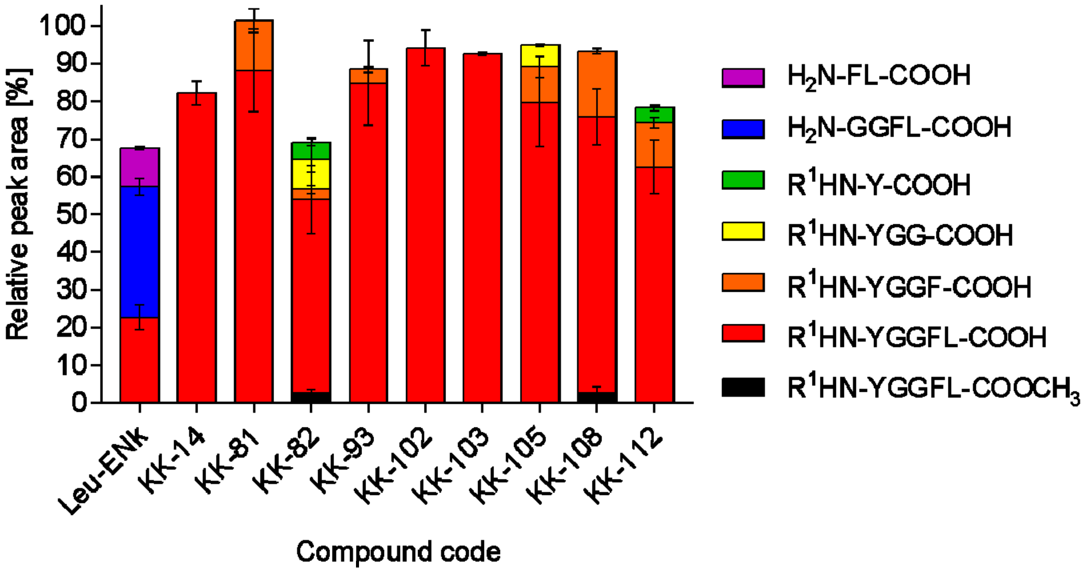

Figure S4. Stability of Leu-ENK analogs after incubation in mouse plasma for 1 h and detected metabolites. Samples were separated by UPLC and peptides were detected by their absorbance at 214 nm. Quantification relied on the peak areas relative to the initial amount of peptide (t = 0 min). Values represent mean ± SEM (n = 3).

Table S1. Binding energy of Leu-ENK and KK-103 to active and inactive delta opioid receptor (DOR) conformations using the molecular mechanics, generalized Born model and solvent accessibility (MM/GBSA) method.

Ligand	State of Receptor	MM/GBSA Binding Free Energy (kcal/mol)	Prediction of Ligand
KK-103	active DOR	−40.33 ± 0.39	Agonist
	inactive DOR	−25.39 ± 3.32	
Leu-ENK	active DOR	−48.58 ± 0.81	Agonist
	inactive DOR	−24.59 ± 0.47	

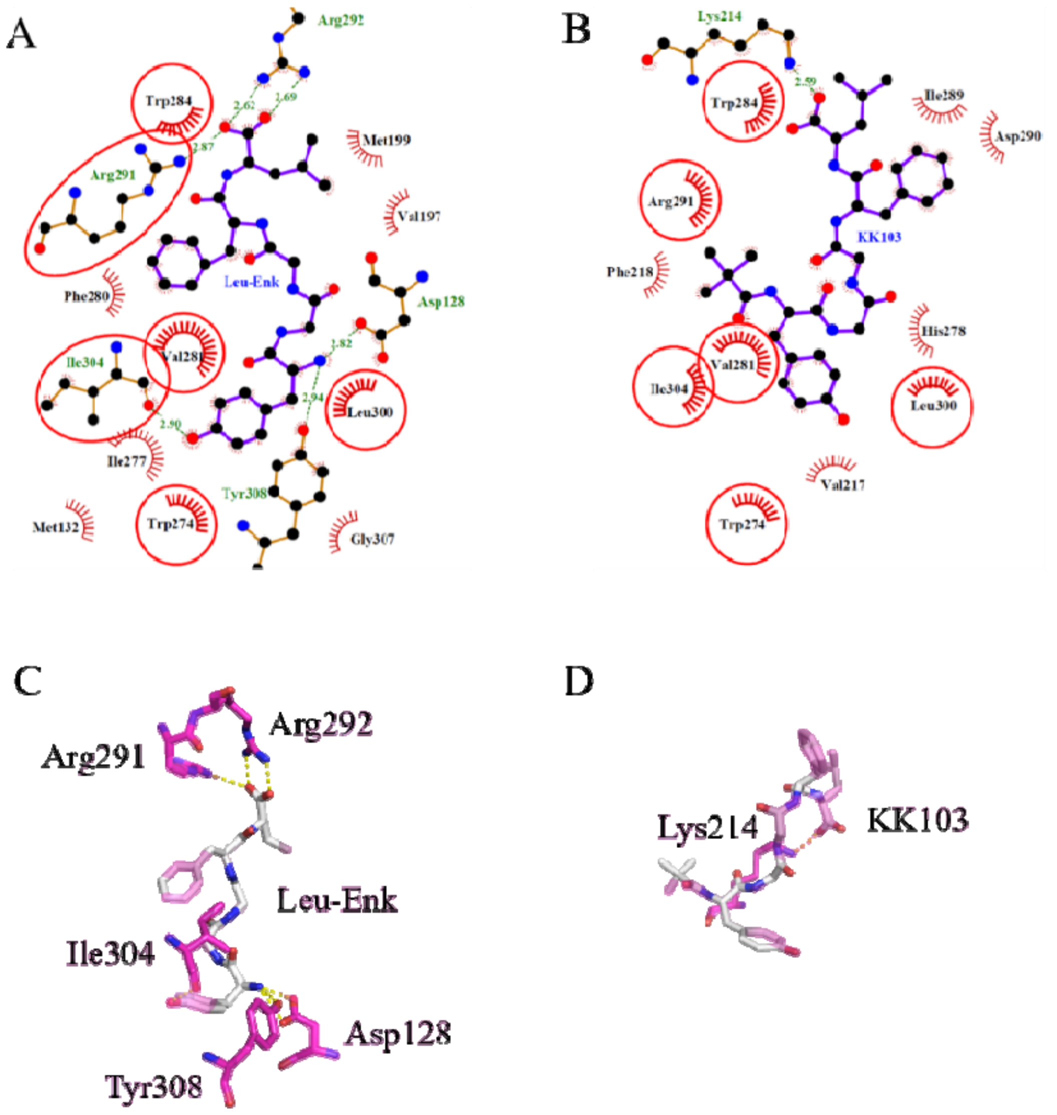

Figure S5. Leu-ENK (**A**, **C**) and KK-103 (**B**, **D**) complexed with active DOR are equilibrated by molecular dynamics simulations (see Supporting Method). Panels A and B are prepared by LigPlot, while C and D are detailed 3D interactions of the active DOR with Leu-ENK and KK-103, respectively.

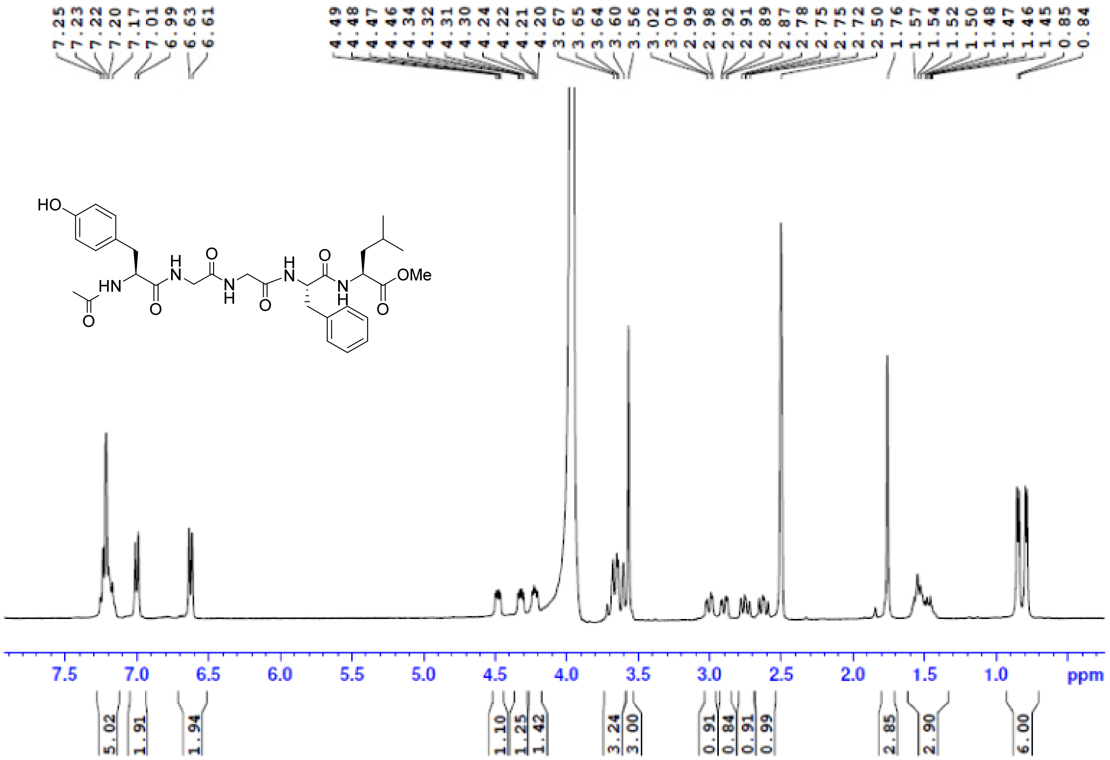

Figure S6. ^{1}H-NMR spectrum of **KK-14.**

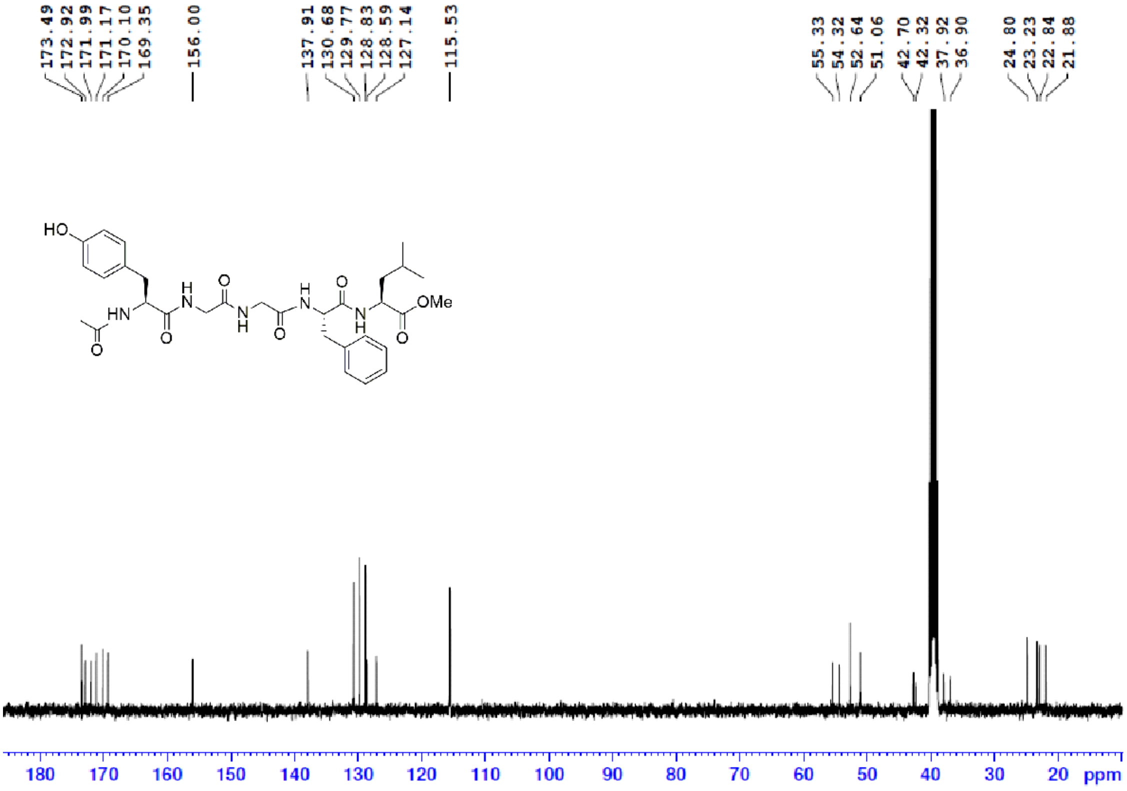

Figure S7. ^{13}C-NMR spectrum of **KK-14.**

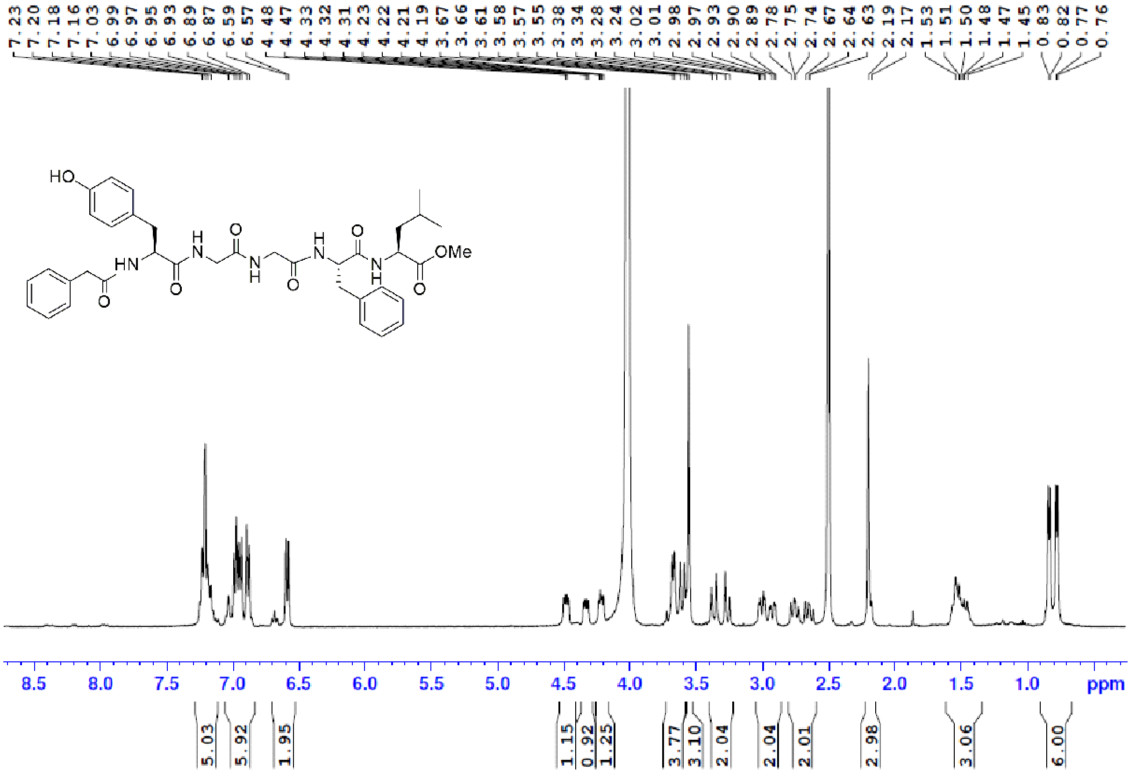

Figure S8. ^{1}H-NMR spectrum of **KK-81.**

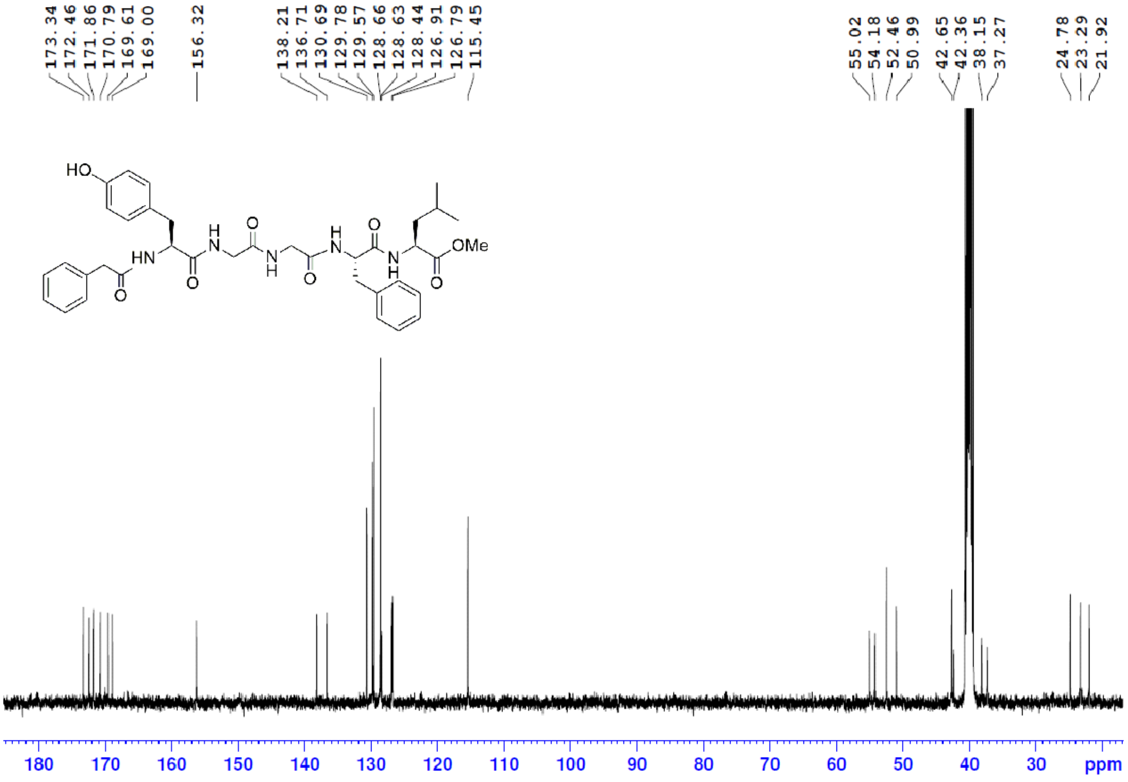

Figure S9. ^{13}C-NMR spectrum of **KK-81.**

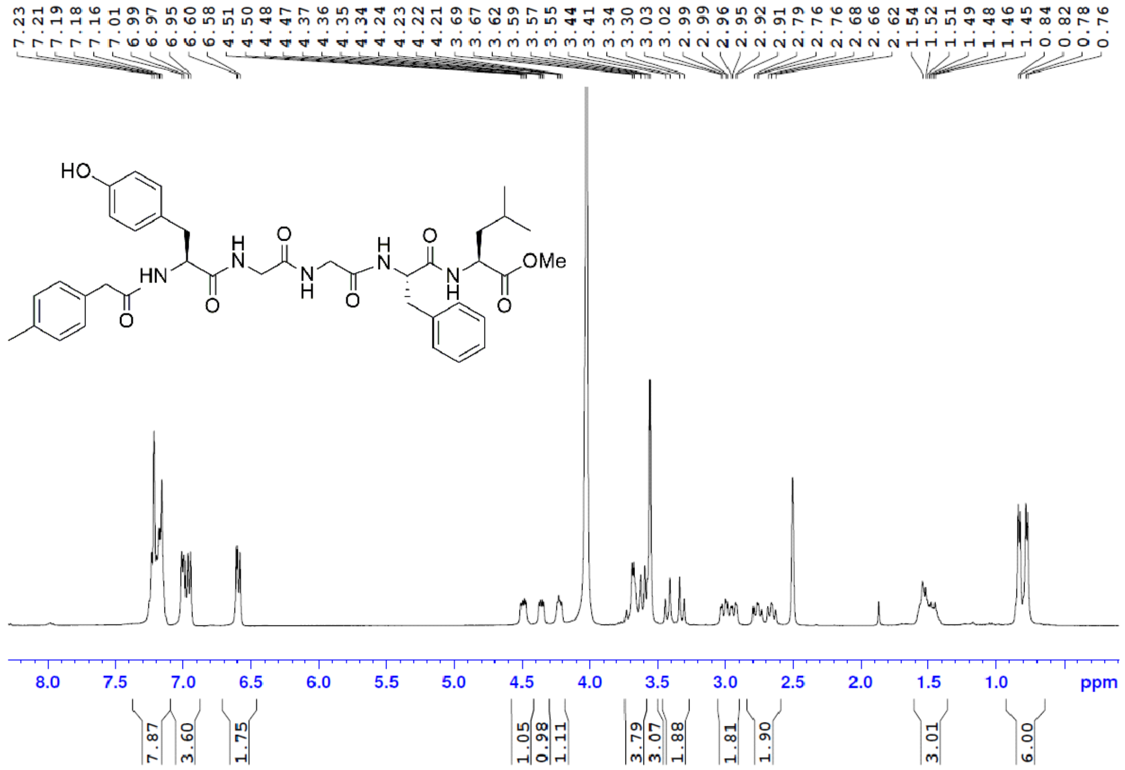

Figure S10. ^{1}H-NMR spectrum of KK-82.

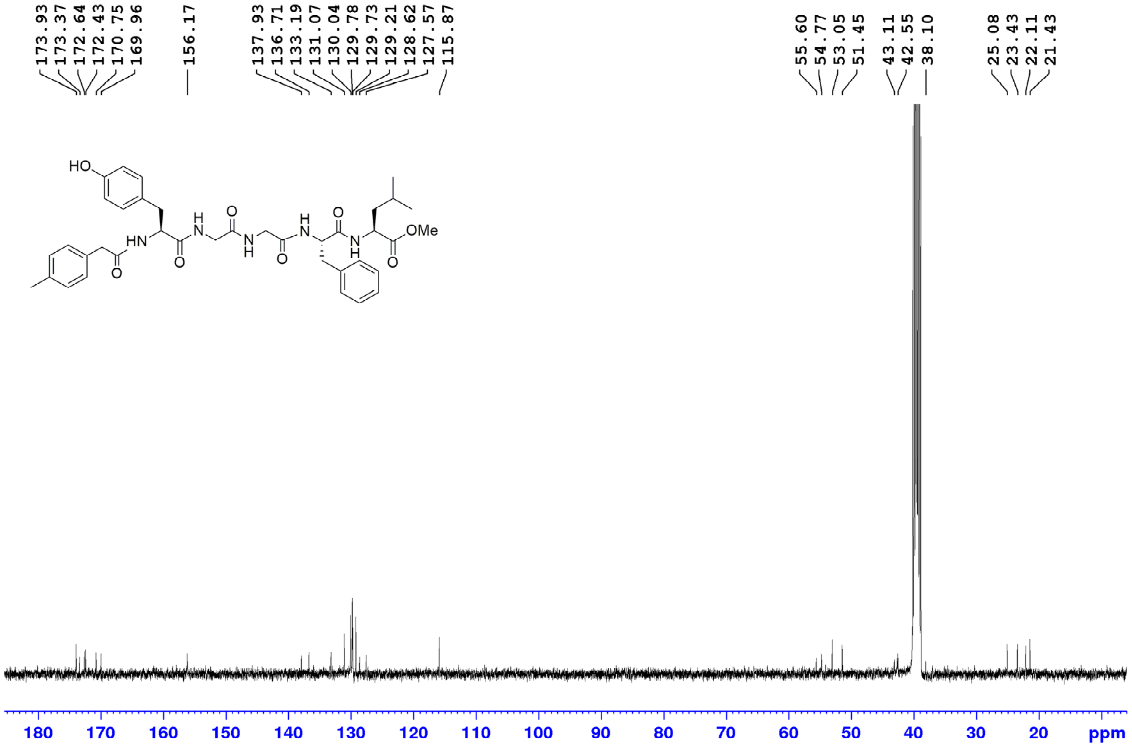

Figure S11. ^{13}C-NMR spectrum of **KK-82.**

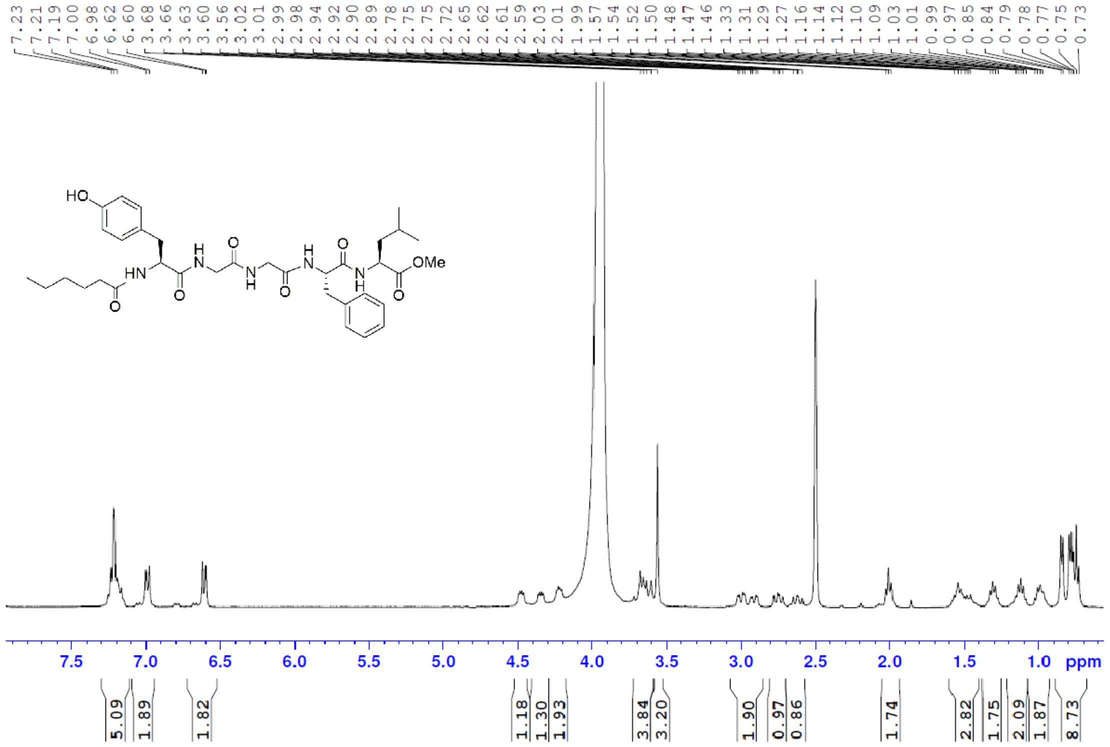

Figure S12. ^{1}H-NMR spectrum of KK-93.

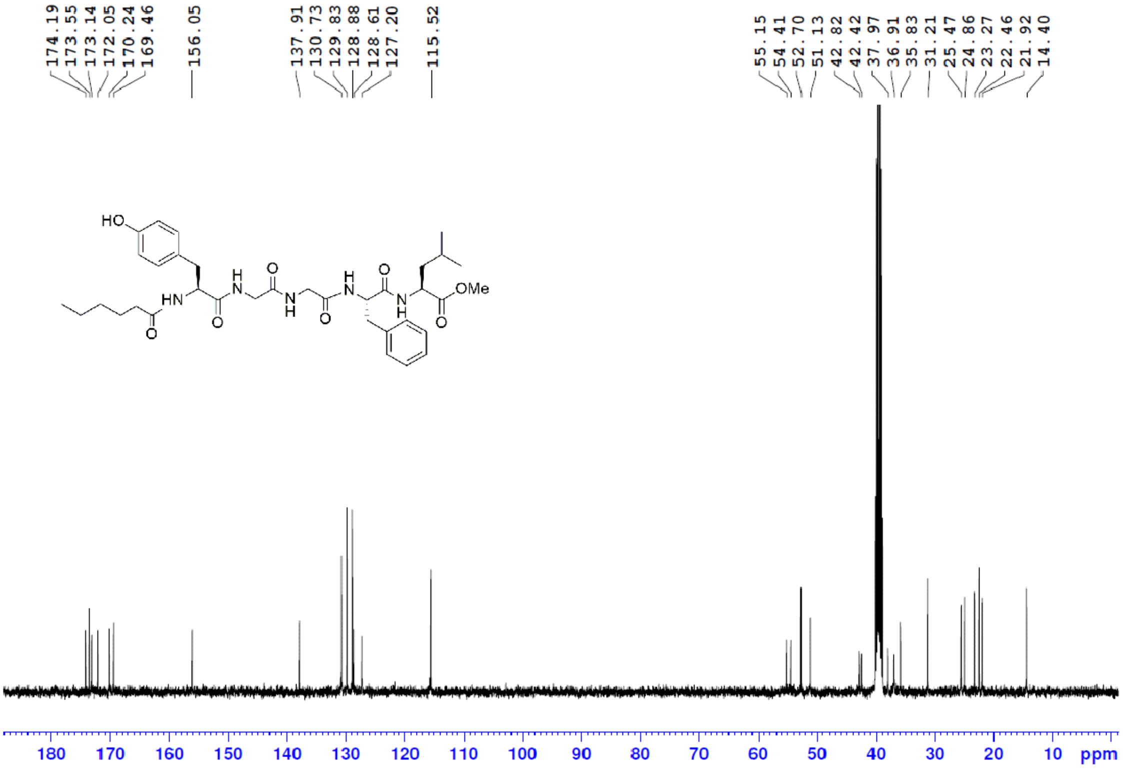

Figure S13. ^{13}C-NMR spectrum of **KK-93.**

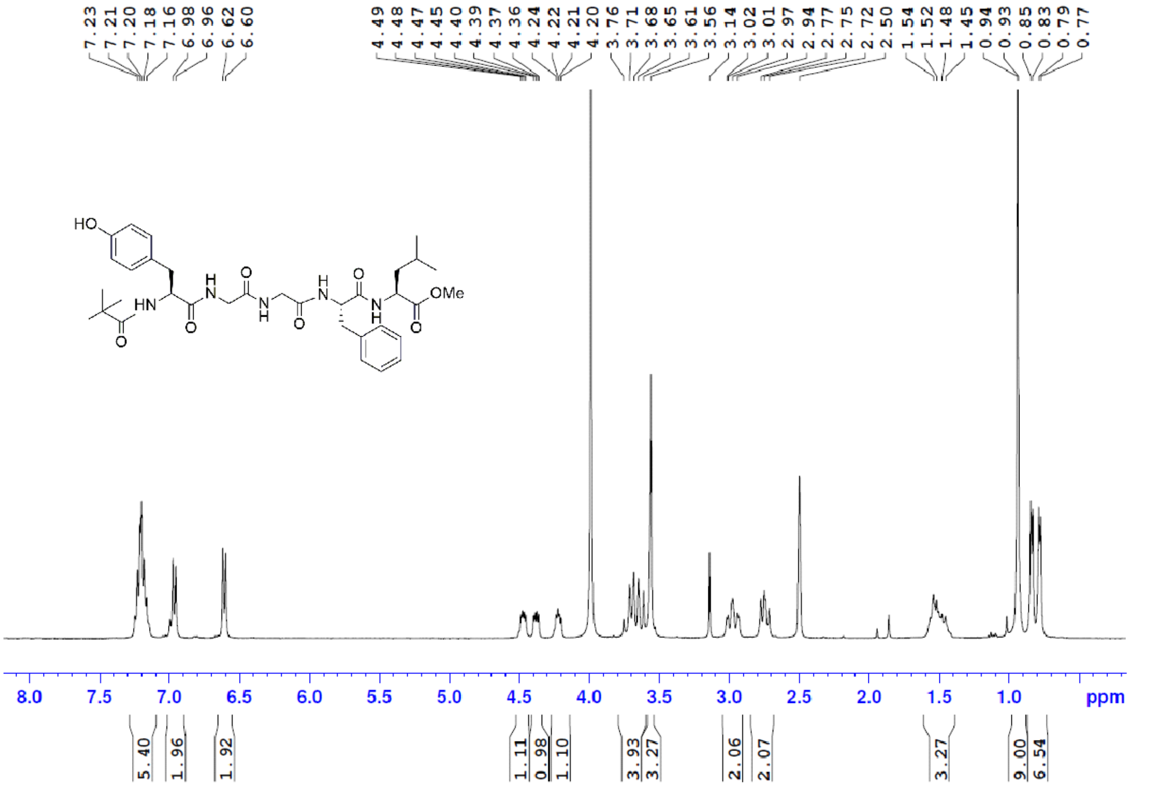

Figure S14. ^{1}H-NMR spectrum of KK-102.

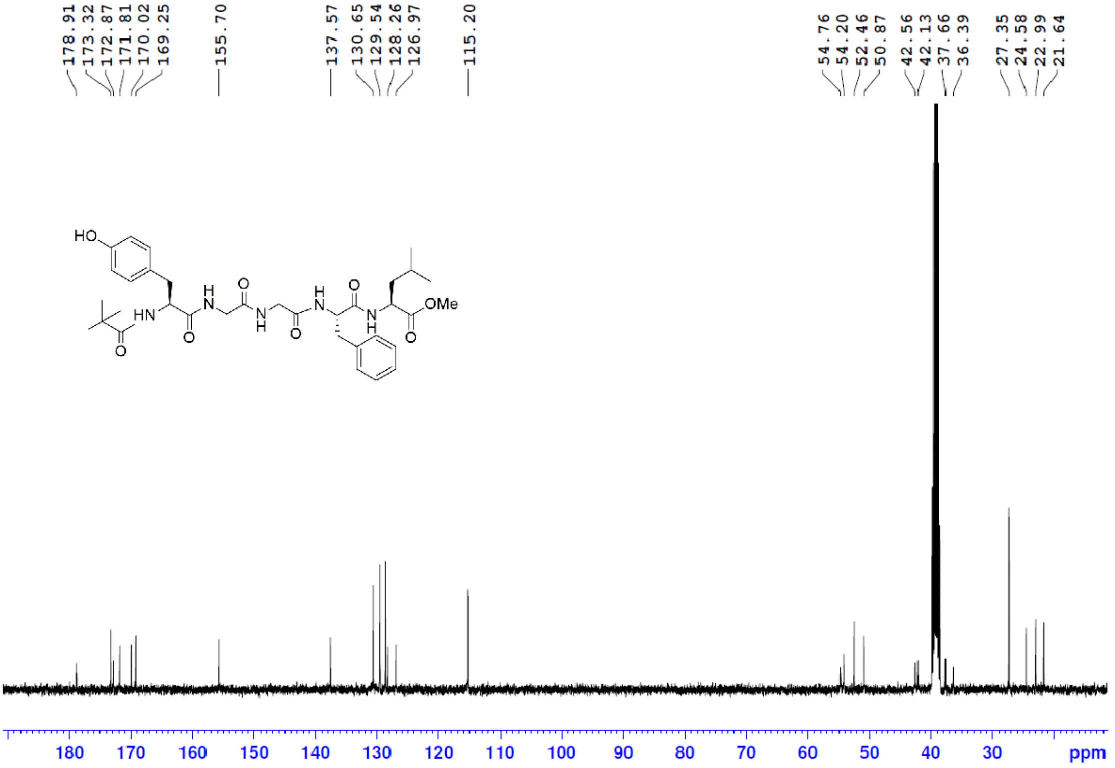

Figure S15. ^{13}C-NMR spectrum of KK-102.

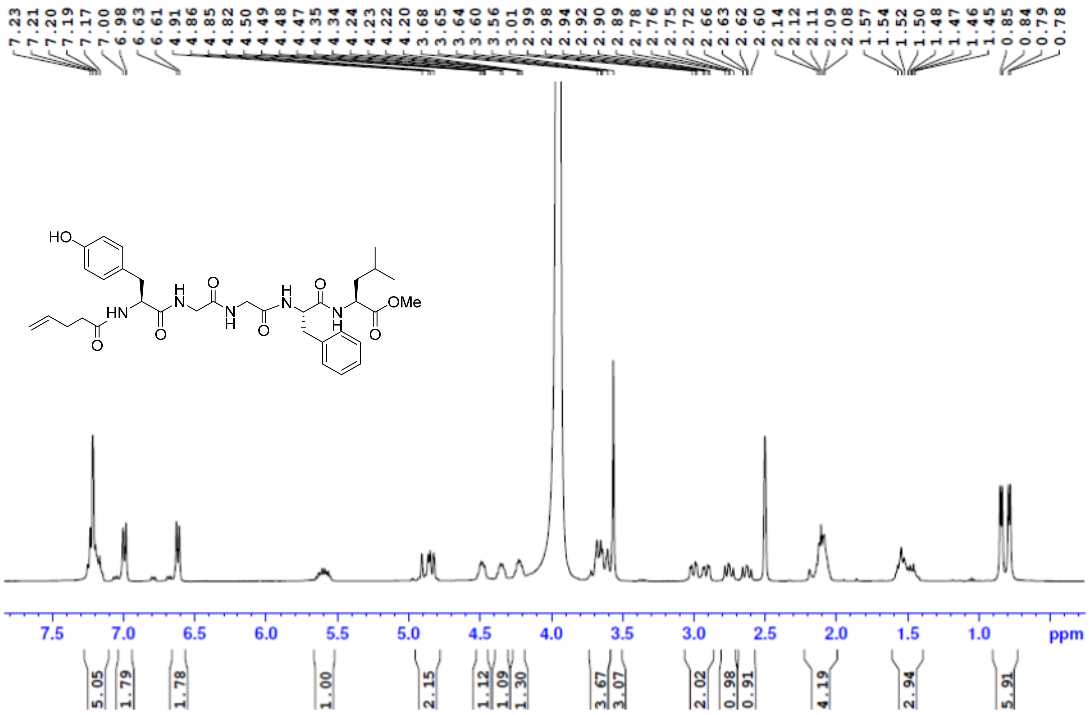

Figure S16. ^{1}H-NMR spectrum of KK-105.

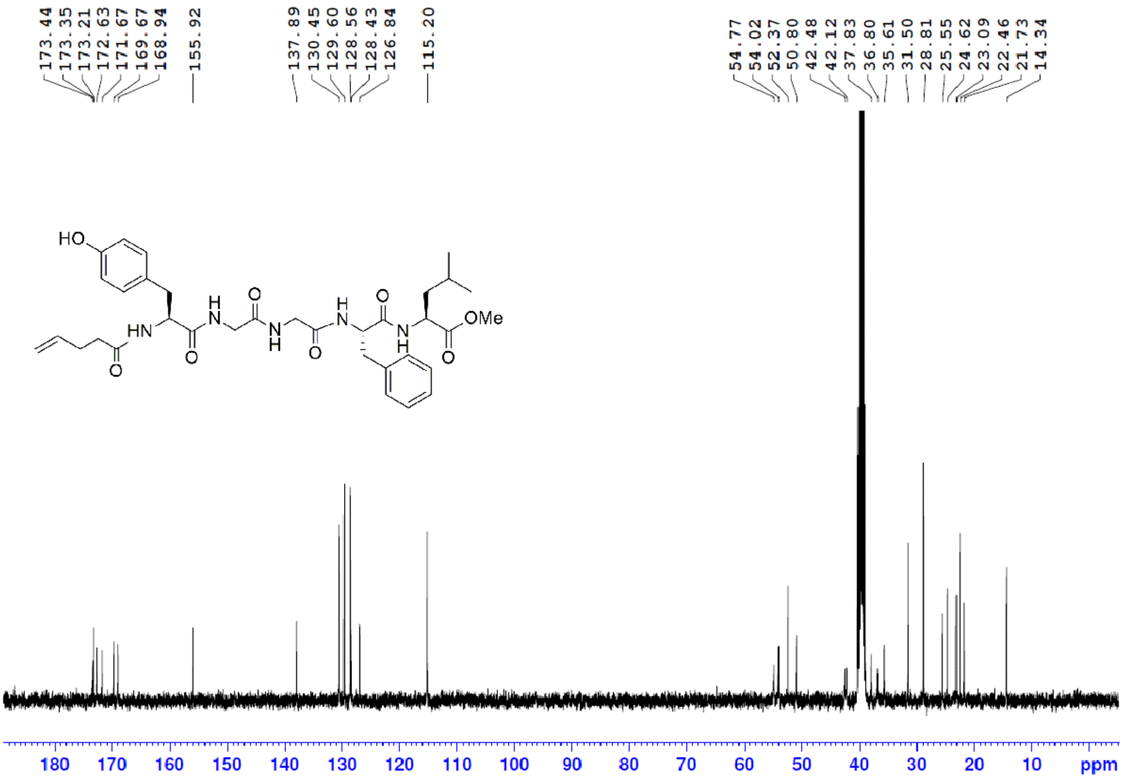

Figure S17. ^{13}C-NMR spectrum of KK-105.

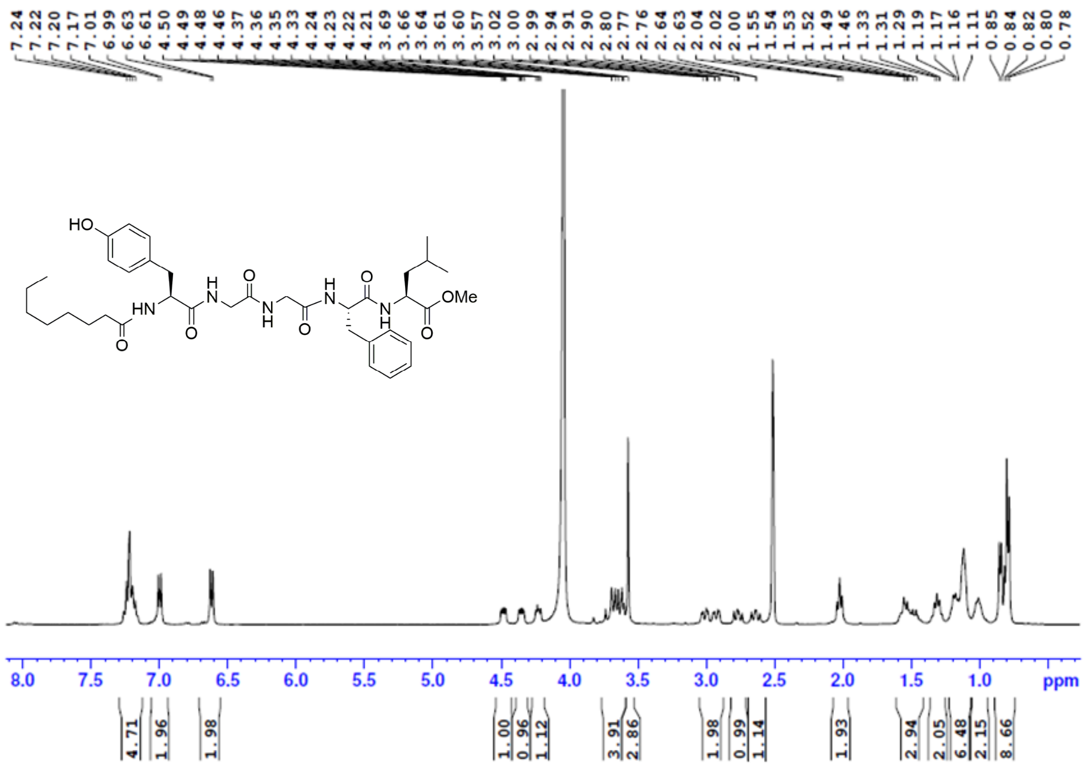

Figure S18. ¹H-NMR spectrum of KK-108.

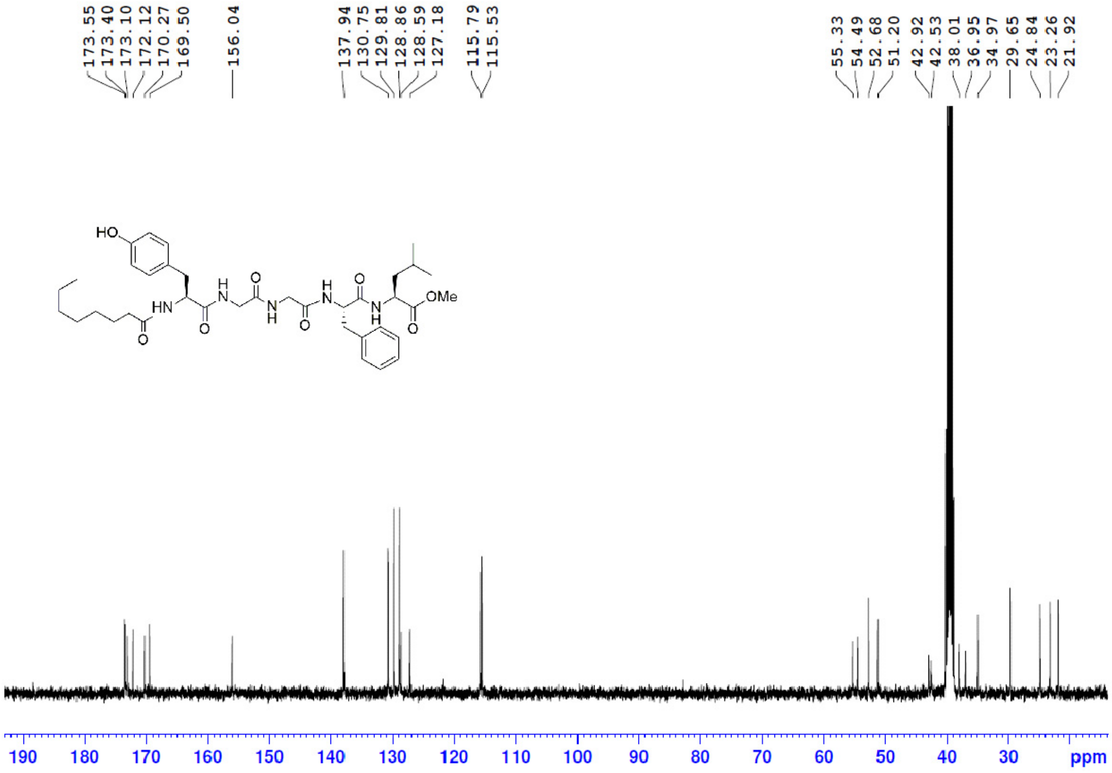

Figure S19. ¹³C-NMR spectrum of KK-108.

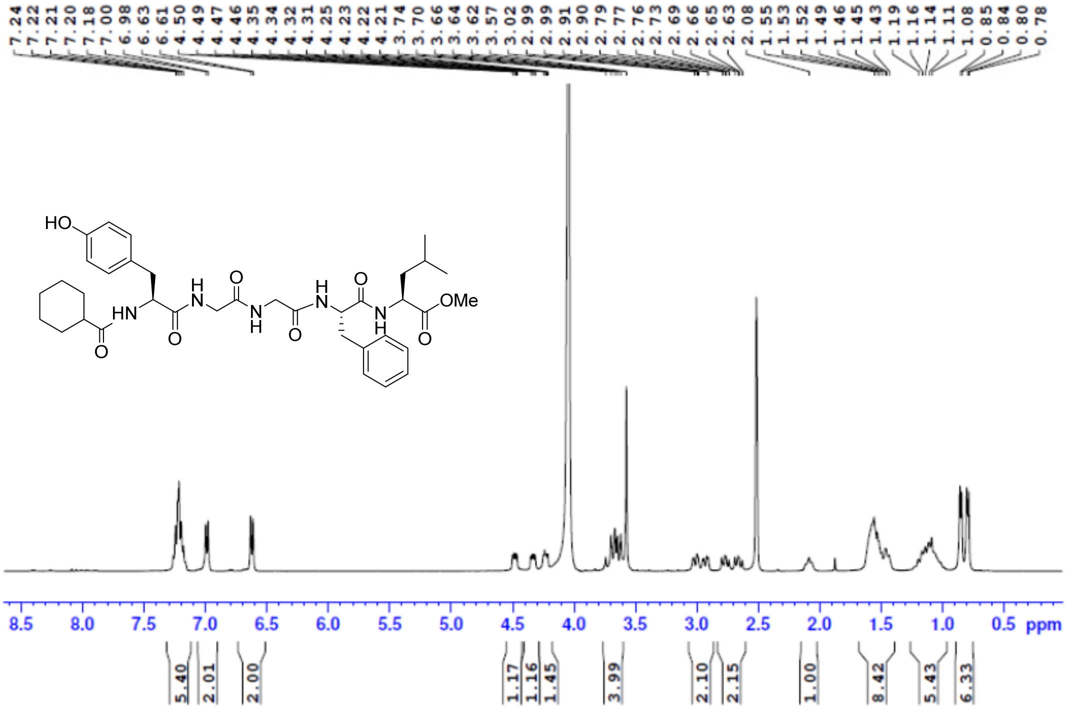

Figure 20. ^{1}H-NMR spectrum of KK-112.

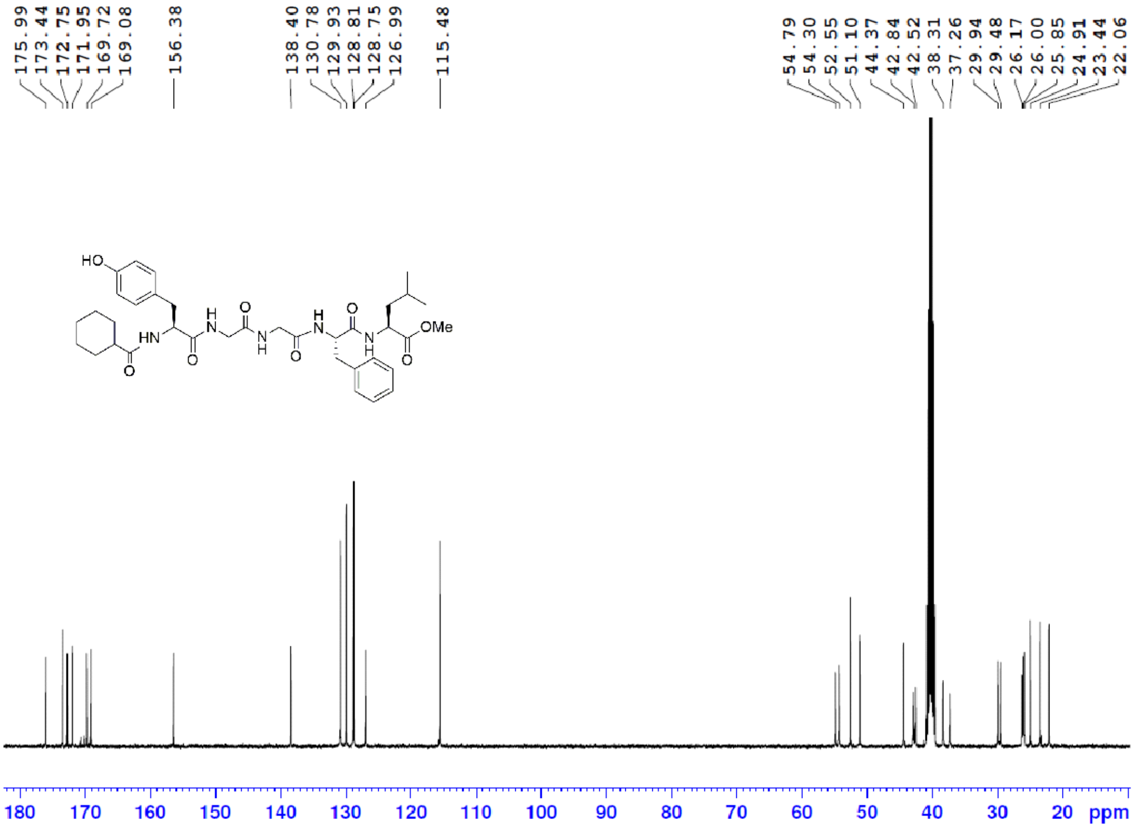

Figure S21. ^{13}C-NMR spectrum of KK-112.

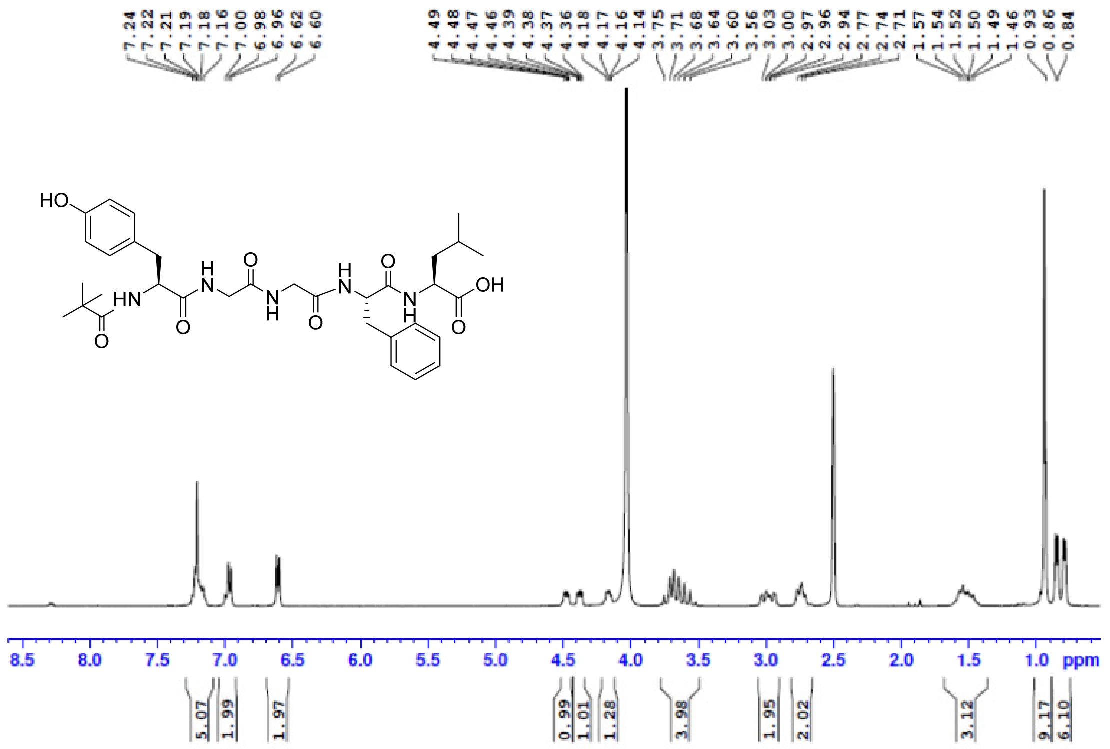

Figure S22. ^{1}H-NMR spectrum of KK-103.

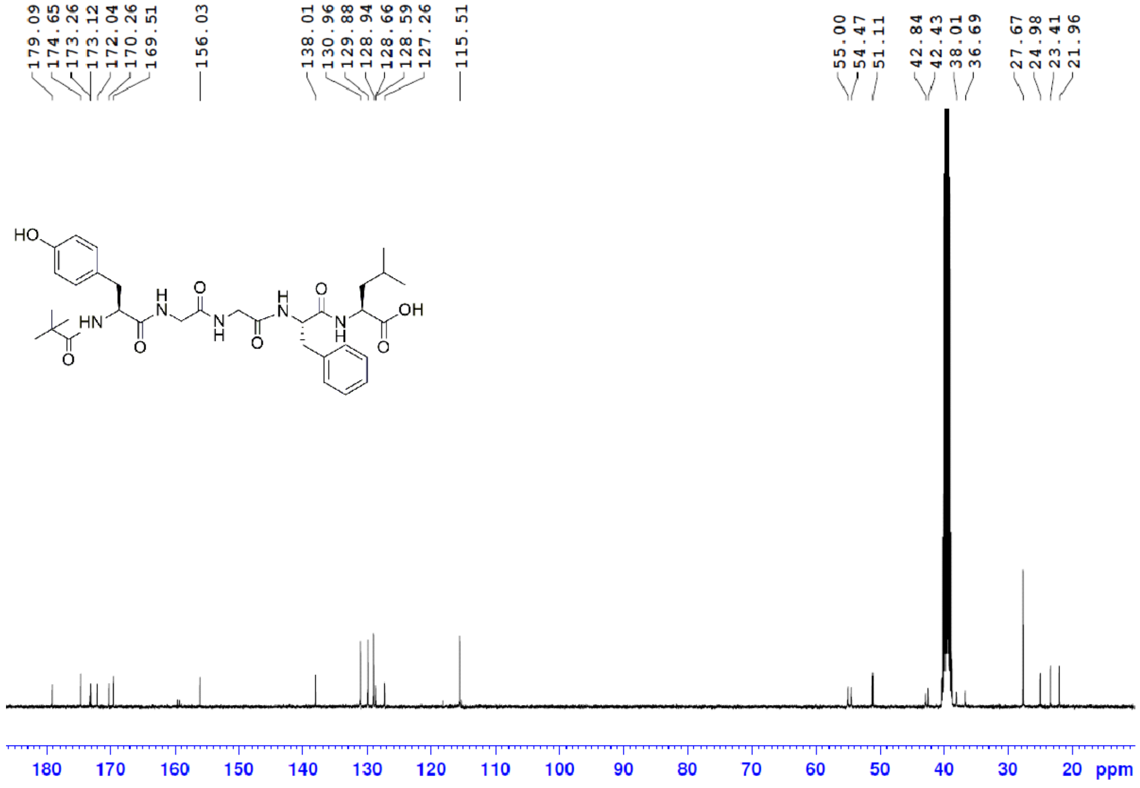

Figure S23. ^{13}C-NMR spectrum of KK-103.

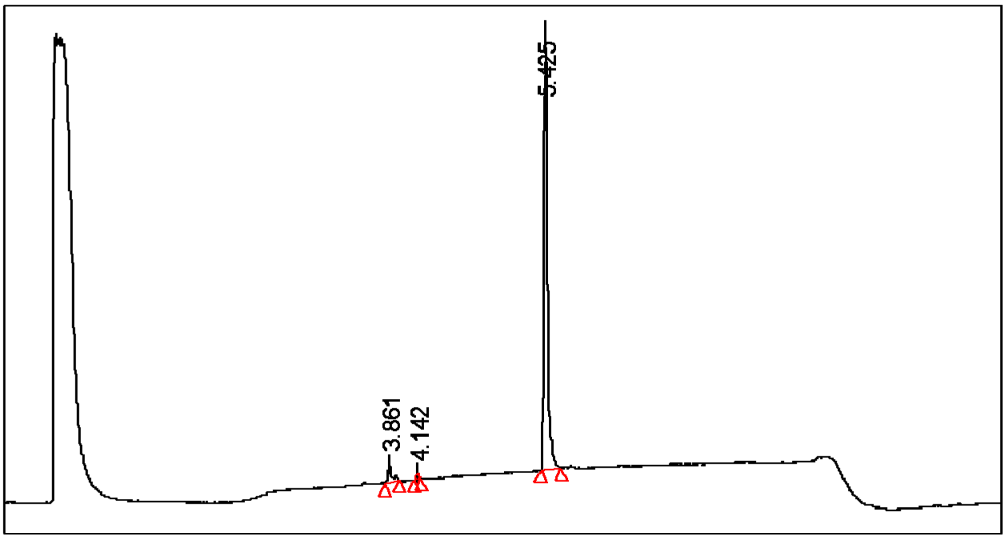

Figure S24. UPLC chromatogram (absorbance at 214 nm) of KK-103 (retention time: 5.4 min). The purity was determined as 95.31%.

1. Supporting Results and Discussion: Molecular Binding of Peptides to DOR

Table S1 shows the binding free energy of the studied compounds (Leu-ENK and KK-103) to both the active and inactive forms of DOR. With a value of −48.58 kcal/mol, Leu-ENK displayed an almost 2-fold lower free binding energy for the active receptor conformation compared to the inactive form (−24.59 kcal/mol), predicting its agonistic effect for DOR. Similarly, KK-103 displayed a lower free binding energy to the active conformation (−40.33 kcal/mol) over the inactive conformation (−25.39 kcal/mol), indicating its agonistic function for DOR.

Figure S5 shows the docking of Leu-ENK and KK-103 into the active form of DOR. The *N*-terminus of Leu-ENK interacts with a highly conserved Asp128 at the cytosolic side of the DOR binding pocket while the *C*-terminal carboxylic acid interacts with Arg291 and Arg292. The phenol group of the N-terminal Tyr forms a hydrogen bond with Ile304's carbonyl group, and the benezene ring forms hydrophobic interaction with Val281. The Phe in Leu-ENK interacts with the proximal Phe280 in the DOR binding pocket and the aliphatic side chain of Arg291.

KK-103 binds DOR in a similar orientation, while the *N*-terminal tertiary butyl group turns away from Asp128 forming a hydrophobic interaction with Phe218 and Arg291. This re-arrangement turns the Phe group away from Phe280 and Arg291 to interact with Ile289 on the opposite side of the binding pocket, while the *C*-terminal carboxylic acid interacts with the positively charged Lys214. Although the tertiary butyl group of KK-103 introduces a favorable hydrophobic interaction, a couple of salt bridges are missing in the DOR-KK103 binding compared to DOR-Leu-ENK. The breakdown of MM/GBSA calculations over MD snapshots reveals that Arg292 and Asp128, among all the DOR interacting residues, most strongly interact with Leu-ENK while interactions with Lys214 and Val281 are strongest with KK-103.

2. Supporting Experimental Methods: Solution-Phase Synthesis of Leu-ENK analogs and Molecular binding

2.1. General Procedure for Peptide Coupling Reaction (Procedure A)

Amino acid coupling was performed according to a previously reported method.[6] To a stirring solution of the carboxyl component (1.0 eq) in dry N-N-dimethylformamide (DMF) at 0 °C, EDC·HCl (1.2 eq) and HOBt (1.2 eq) were subsequentially added. After 10 min, the amine component (1.2 eq) was added to the reaction mixture followed by the addition of triethylamine (3 eq). The mixture was stirred under N_2 atmosphere overnight at r.t. The end of the reaction was monitored by TLC. Afterward, the reaction was quenched with saturated $NaHCO_3$ solution and the mixture was extracted with ethyl acetate (EtOAc). The organic layer was washed with distilled water, 2N HCl solution and saturated NaCl. The organic layer was then dried over anhydrous Na_2SO_4 and the EtOAc evaporated to give the crude peptide.

2.2. General Procedure for Deprotection of Boc (Procedure B)

The removal of Boc was performed according to a previously reported method.[6] The Boc intermediate (1 eq) was dissolved in dry DCM (20 mL) and cooled to 0 °C. TFA (9 eq) was added dropwise and the reaction was stirred under N_2 atmosphere for 1 h at r.t. The TFA was then evaporated and the product precipitated with diethyl ether:*n*-hexane (2:1, *v*/*v*). The precipitate was washed five times with diethyl ether:*n*-hexane (2:1, *v*/*v*), and then dried under vacuum.

2.3. General Procedure for Saponification of the Ester (Procedure C)

To a flask containing the ester (1 eq) in THF:H_2O (2:1, *v*/*v*), LiOH (6 eq) was added. The mixture was stirred at r.t. for 1 h and the progress of saponification was monitored by TLC. The pH of the aqueous layer was adjusted to 2-3 using 2N HCl. The aqueous phase was extracted with EtOAc. Combined organic extracts were dried with Na_2SO_4, filtered and solvents were evaporated under vacuum to give the crude product, which

was purified by silica gel (200 mesh) flash chromatography using methanol/DCM (1:9, *v/v*) to yield the desired saponified intermediate.

(tert-Butoxycarbonyl) glycylglycine (2)

Compound **2** was synthesized according to previous reports with modifications.[7,8] Glycylglycine **1** (3.0 g, 22.5 mmols) was dissolved in 60 mL of dioxane:water (2:1, *v/v*), followed by the addition of 20 mL of 1M NaOH. Di-tert-butyl dicarbonate (5.8 g, 27.0 mmols) was added dropwise and the reaction mixture was stirred at r.t. for 3 h. The solvent was removed under vacuo to about 30–40 mL. The resulting residue was dissolved in 60 mL water and the aqueous solution was acidified using 3M HCl to pH 2–3. The aqueous layer was extracted with EtOAc (3·80 mL). The organic layer was dried over anhydrous Na_2SO_4, and the solvent removed under vacuo. The pure material was obtained as a waxy solid (4.2 g, 80.0%). ^{1}H-NMR (400 MHz, DMSO-d_6): δ 12.55 (br s, 1H, -COOH), 8.05 (br s, 1H, -CO-NH-), 6.99 (t, J = 5.9, 1H, CO-NH), 3.74 (d, J = 5.7 Hz, 2H), 3.55 (d, J = 6.0 Hz, 2H), 1.13 (s, 9H). ESI–MS: calculated exact mass 232.1; found *m/z* 233.0 $[M+H]^+$.

Methyl (tert-butoxycarbonyl)- L-phenylalanyl-L-leucinate (4)

Compound **4** was synthesized according to a previous report with modifications.[9] *N-tert*-Butyloxycarbonyl-L-phenylalanine **3** (4.0 g, 15.0 mmol) was coupled to L-leucine methyl ester **2** (3.4 g, 18.0 mmol) according to procedure A to give the crude dipeptide and was purified by flash chromatography using EtOAc/n-hexane (7:3, *v:v*) to give **4** as a white solid (5.0 g, 75.7%). ^{1}H-NMR (400 MHz, DMSO-d_6): δ 8.27 (d, J = 7.7 Hz, 1H, -CO-NH-), 7.32-7.18 (m, 5H), 6.92 (d, J = 8.7 Hz, 1H, -CO-NH-) 4.35-4.30 (m, 1H), 4.21-4.16 (m, 1H), 3.61 (s, 3H), 2.96-2.92 (m, 1H), 2.75-2.69 (m, 1H), 1.70-149 (m, 4H), 1.29-1-23 (m, 9H), 0.87 (dd, J = 6.4, 23. 3 Hz, 6H). ESI-MS: calculated exact mass 392.5; found *m/z* 393.6 $[M+H]^+$.

Methyl L-phenylalanyl-L-leucinate (5)

Compound **5** was synthesized according to a previous report with modifications.[6] The Boc-dipeptide **4** (5.0 g, 12.7 mmols) was deprotected using procedure B to yield (3.1 g, 83.3%) of **5** as a white solid. ^{1}H-NMR (400 MHz, DMSO-d_6): 8.86 (d, J = 7.7 Hz, 1H, -CO-NH-), 8.17 (br s, 1H, NH), 7.33-7.20 (m, 5H), 4.34 (q, J = 8.2, 14.25, 1H), 4.05 (t, J = 7.0 Hz, 1H), 3.13 -3.09 (m, 1H), 2.96-291 (m, 1H), 1.65-1.52 (m, 3H), 0.88 (dd, J = 6.5, 17.1 Hz, 6H). ESI-MS: calculated exact mass 292.4; found *m/z* 293.4 $[M+H]^+$.

Methyl (tert-butoxycarbonyl)glycylglycyl-L-phenylalanyl-L-leucinate (6)

Compound **6** was synthesized according to a previous report with modifications.[10] Boc-glycylglycine **2** (3.5 g, 15.02 mmols) was coupled to methyl L-phenylalanyl-L-leucinate **5** (4.40 g, 15.02 mmols) according to procedure A to give the crude tetrapeptide and was purified by flash chromatography using EtOAc:*n*-hexane (7:3 *v:v*) to afford **6** as a white solid (4.9 g, 81.1%). ^{1}H-NMR (400 MHz, DMSO-d_6): δ 8.37(d, J = 7.5 Hz, 1H, -CO-NH-), 8.08 (d, J = 8.0 Hz, 1H, -CO-NH-) 7.91 (t, J = 5.1 Hz, 1H, -CO-NH-), 7.26-7.19 (m, 5H), 6.99 (t, J = 5.6 Hz, 1H, -CO-NH-), 4.58-4.52 (m, 1H), 4.31-4.25 (m, 1H), 3.72 (dd, J = 5.7, 16.85 H, 1H), 3.62 (s, 3H), 3.52-3.53 (m,2H), 1.62-1.52 (m,3H), 1.38 (s, 9H), 0.87 (dd, J = 6.1, 22.1 Hz, 6H). ESI-MS: calculated exact mass 506.3; found *m/z* 507.6 $[M+H]^+$.

Methyl glycylglycyl-L-phenylalanyl-L-leucinate (7)

Compound **7** was synthesized according to a previous report with modifications.[11] The Boc-tetrapeptide **6** (4.0 g, 7.9 mmols) was deprotected with TFA (5.4 mL, 71.1 mmols) using procedure B to yield **7** as a white solid (2.9 g, 90.6%). ^{1}H-NMR (400 MHz, DMSO-d_6): δ 8.48 (d, *J* = 6.7 Hz, 1H, -CO-NH-), 8.27 (d, *J* = 8.4 Hz, 1H, -CO-NH-), 8.00 (s, 2H -NH), 7.27-7.18 (m, 5H), 4.61-4.56 (m, 1H), 4.31-4.26 (m, 1H), 3.84

(dd, *J* = 5.4, 16.8 Hz, 1H), 3.62 (s, 3H), 2.76-2.70 (m, 1H), 1.63-1.48 (m, 3H), 0.88 (dd, *J* = 6.2, 22.1Hz, 6H). ESI-MS: calculated exact mass 406.2; found *m/z* 407.2 $[M+H]^+$.

Pivaloyl-L-tyrosine (8)

Pivalic acid (0.4 g, 3.91 mmol) was reacted with methyl L-tyrosinate (0.76, 3.91 mmol) according to procedure A to crude oil. The reaction progress was monitored by TLC (EtOAc/*n*-hexane, 6:4, *v/v*) and mass spectrometry was performed to confirm the methyl pivaloyl-L-tyrosinate. Without further purifications, the crude oil (0.7 g, 2.4 mmol) was suspended in THF (7 mL) and added to a solution of LiOH (0.32 g, 14.4 mmol) in H_2O (3 mL) according to procedure C to give the crude product, which was purified by flash chromatography using methanol/DCM (1:9, *v/v*) to yield compound **8** as a colorless oil (0.45 g, 68.1%). ^{1}H-NMR (400 MHz, deuterated dimethyl sulfoxide, DMSO-d_6) δ 12.41 (br s, 1H, -COOH), 9.17 (br s, 1H, -OH), 7.42 (d, *J* = 8.0 Hz, 1H, -CO-NH-), 7.00 (d, *J* = 8.2 Hz, 2H), 6.62 (d, *J* = 8.1 Hz, 2H), 4.33-4.28 (m, 1H), 2.94 (dd, *J* = 5.1, 13.9 Hz, 1H), 2.86-2.80 (m 1H), 1.01 (s, 9H). ESI-MS: calculated exact mass 265.1; found *m/z* 266.1 $[M+H]^+$ and 288.1 $[M+Na]^+$.

(2-Phenylacetyl)-L-tyrosine (9)

Compound **9** was synthesized according to a previous report with modifications.[12] Phenyl acetic acid (0.4 g, 1.5 mmols) was reacted with methyl L-tyrosinate (0.29 g, 1.5 mmols) according to procedure A to yield a crude solid. Without further purification, the crude intermediate (0.69 g, 2.1 mmol) was reacted with LiOH (0.27 g, 13.1 mmol) according to procedure C to yield compound **9** as a white solid (0.44 g 61.1%). %). ^{1}H-NMR (400 MHz, DMSO-d_6) *δ* 12.40 (br s, 1H, -COOH), 9.12 (br s, 1H, -OH), 7.39 (d, *J* = 8.1 Hz, 1H, -CO-NH-), 7.24 -7.02 (m, 5H), 6.90 (d, *J* = 8.1 Hz, 2H), 6.73 (d, *J* = 8.2 Hz, 2H), 4.34-4.27 (m, 1H), 3.57-3.45 (m, 2H) 3.16-2.94 (m, 2H). ESI-MS: calculated exact mass 299.1; found *m/z* 300.4 $[M+H]^+$ and 322.1 [M+Na]+.

2-(p-Tolyl)acetyl)-L-tyrosine (10)

2-(*p*-tolyl)acetic acid (0.4 g, 2.64 mmols) was reacted with methyl L-tyrosinate (0.51 g, 2.64 mmols) according to procedure A to yield a crude solid. Without further purification, the crude intermediate (0.7 g, 2.12 mmol) was reacted with LiOH according to procedure C to yield compound **10** as a white solid (0.42 g, 58.3%). ^{1}H-NMR (400 MHz, DMSO-d_6) *δ* 12.45 (br s, 1H, -COOH), 9.06 (br s, 1H, -OH), 8.33 (br s, 1H, -CO-NH), 7.38-7.33 (m, 4H), 7.08-6.95 (m, 4H), 4,35 (m, 1H), 3.54-3.45 (m, 2H), 3.14-2.91 (m, 2H), 2.25 (s, 3H). ESI-MS: calculated exact mass 313.1; found *m/z* 314.1 $[M+H]^+$.

Hexanoyl-L-tyrosine (11)

Compound **11** was synthesized according to a previous report with modifications.[13] Hexanoic acid (0.4 g, 3.44 mmols) was reacted with methyl L-tyrosinate (0.67, 3.44 mmols) according to procedure A to yield a light yellow solid. Without further purification, the crude intermediate (0.67 g, 2.16 mmol) was reacted with LiOH (0.27 g, 12.96 mmol) according to procedure C to yield compound **11** as a white solid (0.42 g, 70.0%). ^{1}H-NMR (400 MHz, DMSO-d$_6$) δ 12.45 (br s, 1H, -COOH), 9.17 (br s, 1H, -OH), 8.05 (d, J = 8.1 Hz, 1H, -CO-NH-), 6.99 (d, J = 8.2 Hz, 2H), 6.63 (d, J = 8.2 Hz, 2H), 4.35-4.29 (m, 1H), 2.91 (dd, J = 4.6, 13.9 Hz, 1H), 2.73-2.67 (m, 1H), 2.08-1.98 (m, 2H), 1.43-1.35 (m, 2H), 1.26-1.08(m, 4H), 0.82 (t, J = 7.0 Hz, 3H) ESI-MS: m/z $[M + H]^+$. ESI–MS: calculated exact mass 279.1; found *m/z* 280.2 $[M+H]^+$.

Octanoyl- L-tyrosine (12)

Compound **12** was synthesized according to a previous report with modifications.[13] Octanoic acid (0.4 g, 1.29 mmols) was reacted with methyl L-tyrosinate (0.25 g, 1.29 mmols) according to procedure A to yield a colorless solid. Without further purification, the crude intermediate (0.72 g, 2.22 mmol) was reacted with LiOH (0.16 g, 7.78 mmol) according to procedure C to yield compound **12** as a

white solid (0.41 g, 61.7%). ^{1}H-NMR (400 MHz, DMSO-d$_6$) δ 12.44 (br s, 1H, -COOH), 9.16 (br s, 1H, -OH), 8.03 (d, J = 8.1 Hz. 1H, -CO-NH-), 6.97 (d, J = 8.1 Hz, 2H), 6.62 (d, J = 8.1 Hz, 2H), 4.35-4.27 (m, 1H), 2.93 (m, 1H), 2.70-2.65 (m,1H), 2.15 (t, J = 7.4 Hz, 2H), 2.06-1.97 (m, 2H),1.50-1.46 (m, 2H), 1.30-1.27 (m, 6H), 0.86-0.88 (m, 3H). ESI-MS: calculated exact mass 307.2; found *m*/*z* 308.2 $[M+H]^+$.

Penta-4-enoyl-L-tyrosine (13)

Compound **13** was synthesized according to a previous report with modifications.[14] Pentenoic acid (0.4 g, 2.77 mmols) was reacted with methyl L-tyrosinate (0.51 g, 2.77 mmols) according to procedure A to yield a white solid. Without further purification, the crude residue (0.66 g, 2.35 mmol) was reacted with LiOH (0.29 g, 14.14 mmol) according to procedure C to yield compound **13** as a white solid (0.41 g, 66.1%). ^{1}H-NMR (400 MHz, DMSO-d$_6$) δ 12.42 (br s, 1H, -COOH), 9.15 (br s, 1H, -OH), 7.55 (br s, 1H, -CO-NH-), 7.15 (d, J = 8.4 Hz, 2H), 6.30, (d, J = 8.4 Hz, 2H), 5.80-5.70 (m, 1H), 5.15-4.89 (m, 2H), 4.40 (q, J = 5.1, 8.15 Hz, 1H), 3.20-3.15 (m, 2H), 2.95 (dd, J = 5.1, 13.94 Hz, 1H) 2.85-2.79 (m, 1H), 2.31-2.40 (m, 2H). ESI-MS: calculated exact mass 263.1; found *m*/*z* 264.7 $[M+H]^+$.

(Cyclohexane carbonyl)- L-tyrosine (14)

Compound **14** was synthesized according to a previous report with modifications.[15] Cyclohexonic acid (0.4 g, 3.12 mmols) was reacted with methyl L-tyrosinate (0.60 g, 3.12 mmols) according to procedure A to yield a white solid. Without further purification, the crude residue (0.75 g, 2.45 mmol) was reacted with LiOH according to procedure C to yield compound **14** as a white solid (0.41 g, 58.5%). ^{1}H-NMR (400 MHz, DMSO-d$_6$) δ 12.09 (br s, 1H -COOH), 9.14 (br s, 1H, -OH), 8.00 (br s, 1H, -CO-NH-), 6.99 (d, J = 8.5, Hz, 2H), 6.76 (d, J = 8.3 Hz, 2H), 4.30-4.24 (m, 1H), 2.92 (m, 1H), 2.80-2.72 (m 1H) 2.38-2.36 (m, 1H), 1.73-1.75 (m, 2H) 1.70-1.43 (m, 8H). ESI-MS: calculated exact mass 291.1; found *m*/*z* 292.2 $[M+H]^+$.

Methyl acetyl-L-tyrosylglycylglycyl-L-phenylalanyl-L-leucinate (KK-14)

Commercially available acetyl-L-tyrosine (0.2 g, 0.89 mmol) was reacted with methyl glycylglycyl-L-phenylalanyl-L-leucinate **7** (0.36 g, 0.89 mmol) according to procedure A to yield the crude residue and was purified by silica gel flash chromatography eluting with methanol/DCM (1:9, *v*/*v*) to yield KK-14 as white solid (0.40 g, 74.0%). ^{1}H-NMR (400 MHz, 8:2 DMSO-d_6:H_2O): δ 7.25-7.16 (m, 5H), 6.98 (d, *J* = 7.9 Hz, 2H), 6.62 (d, *J* = 8.0 Hz, 2H), 4.47 (q, *J* = 3.3, 9.5 Hz, 1H), 4.31 (q, *J* = 4.4, 9.5, 1H), 4.23-4.20 (m, 1H) 3.67-3.56 (m, 3H), 3.56 (s, 3H), 3.00 (dd, *J* = 4.0, 13.8, 1H), 2.89 (dd, *J* = 4.4,, 14.0 m, 1H), 2.77-2.70 (m, 1H), 2.65-02.59 (m, H), 1.75 (s, 3H), 1.56-1.44 (m, 3H), 0.81 (dd, *J* = 5.8, 22.8 Hz, 6H). ^{13}C NMR (200 MHz, 8:2 DMSO-d_6:H_2O) δ 173.4, 172.9, 171.9, 171.17, 170.10, 169.3, 156.0, 137.9, 130.6, 129.7, 128.8, 128.5, 127.1, 115.3, 55.3, 54.3, 52.6, 51.0, 42.7, 42.3, 37.9, 36.9, 24.8, 23.2, 22.8, 21.8. UPLC: retention time = 7.43 min. ESI-MS: calculated exact mass 611.3; found *m*/*z* 612.6 $[M+H]^+$.

Methyl (2-phenylacetyl)-L-tyrosylglycylglycyl-L-phenylalanyl-L-leucinate (KK-81)

Phenylacetyl-L-tyrosine **9** (0.3 g, 1.0 mmol) was reacted with methyl glycylglycyl-L-phenylalanyl-L-leucinate **7** (0.40 g, 1.0 mmol) according to procedure A to yield crude residue and was purified by flash chromatography eluting with methanol/DCM (1:9, *v*/*v*) to yield KK-81 as a white solid (0.41 g, 60.0%). ^{1}H-NMR (400 MHz, 8:2 DMSO-d_6:H_2O) δ 7.23-7.16 (m, 8H), 7.00-6.94 (m, 4H), 6.59 (d, *J* = 8.7 Hz, 2H), 4.48 (q, *J* = 4.1, 9.09 Hz,1H), 4.35 (q, *J* = 4.3, 9.4, Hz, 1H), 4.23-4.20 (m, 1H), 3.72-3.55 (m, 4H), 3.63 (s, 3H), 3.44-3.30 (m, 2H), 3.02-2.91 (m, 2H), 2.78-2.62 (m, 2H), 1.67-1.54-1.45 (m, 3H) 0.80 (dd, *J* = 5.6, 23.2 Hz, 9H). ^{13}C-NMR (200 MHz, 8:2 DMSO-d_6:H_2O) δ 173.3, 172.4, 171.8, 170.7, 169.6, 169.0, 156.3, 138.2, 136.7, 130.6, 129.7, 129.5, 128.66, 128.63, 128.4, 126.9, 126.7, 115.4, 55.0, 54.1, 52.4, 50.9, 42.6, 42.3, 38.1, 37.2, 24.7, 23.2, 21.9. UPLC: retention time = 7.80 min. ESI-MS: calculated exact mass 687.3; found *m*/*z* 688.3 $[M+H]^+$.

Methyl (2-(p-tolyl)acetyl)-L-tyrosylglycylglycyl-L-phenylalanyl-L-leucinate (KK-82)

2-(*p*-Tolyl)acetyl)-L-tyrosine **10** (0.3 g, 0.95 mmol) was reacted with methyl glycylglycyl-L-phenylalanyl-L-leucinate **7** (0.38 g, 0.95 mmol) according to procedure A to yield the crude residue and was purified by flash chromatography eluting with methanol/DCM (1:9, *v*/*v*) to yield KK-82 as a white solid (0.4 g, 59.7%). ^{1}H-NMR (400 MHz, 8:2 DMSO-d_6:H_2O) δ 7.25-7.16 (m, 5H) 7.03-6.87 (m, 6H), 6.59-6.57 (m, 2H), 4.47.(q, *J* = 4.3, 9.43 Hz, 1H), 4.31 (q, *J* = 4.3, 9.3 Hz, 1H) 4.23-4.19 (m, 1H), 3.67-3.57 (m, 4H), 3.55 (s, 3H), 3.38-3.24 (m, 2H), 3.02-2.89 (m, 2H), 2.78-2.72 (m, 1H), 2.67-2.61 (m,1H) 2.22 (s, 3H) 1.66-1.54 (m, 3H) 0.88 (dd, *J* = 5.6, 23.3 Hz,6H); ^{13}C-NMR (200 MHz, 8:2 DMSO-d_6:H_2O) δ 173.9, 173.3, 172.6, 172.4, 170.7, 169.9, 156.1, 137.9, 136.7, 133.1, 131.0, 130.0, 129.78, 129.73, 129.2, 128.6, 127.5, 115.8, 55.6, 54.7, 53.0, 51.45, 43.11, 42.5, 38.1, 25.0, 23.4, 22.1, 21.4. UPLC: retention time = 8.08 min. ESI-MS: calculated exact mass 701.3; found *m*/*z* 702.3 $[M+H]^+$.

Methyl hexanoyl-L-tyrosylglycylglycyl-L-phenylalanyl-L-leucinate (KK-93)

Hexanoyl-L-tyrosine 11 (0.3 g, 1.0 mmol) was reacted with methyl glycylglycyl-L-phenylalanyl-L-leucinate **7** (0.43 g, 1.0 mmol) according to procedure A to yield crude residue and was purified by flash chromatography eluting with methanol/DCM (1:9, *v*/*v*) to yield KK-93 as a white solid (0.41 g, 57.3%). ^{1}H-NMR (400 MHz, 8:2 DMSO-d_6:H_2O) δ 7.23-7.16 (m, 5H), 6.98 (d, *J* = 8.2 Hz, 2H), 6.60, (d, *J* = 8.2 Hz, 2H), 4.47 (q, *J* = 4.0, 8.9 Hz, 1H), 4.50-4.41 (q, *J* = 4.3, 9.8 1H), 4.22-4.20 (m, 2H), 3.72-3.60 (m, 4H), 3.65 (s, 3H), 3.02-2.89 (m, 2H), 2.77-2.72 (m, 1H), 2.64-2.58 (m, 1H) 2.00 (t, *J* = 7.2 Hz, 2H), 1.56-1.45 (m, 3H), 1.32-1.27 (m, 2H), 1.15-1.08 (m, 2H), 1.02-0.97 (m, 2H), 0.85-0.72 (m, 9H); ^{13}C-NMR (200 MHz, 8:2 DMSO-d_6:H_2O) δ 174.1, 173.5, 173.1, 172.0, 170.2, 169.4, 156.0, 137.9, 130.7, 129.8, 128.8, 128.6, 127.2, 115.5, 55.1, 54.4, 52.7, 51.1, 42.8, 42.4, 37.9, 36.9, 35.8, 31.2, 25.4, 24.8, 23.2, 22.4, 21.9, 14.4. UPLC: retention time = 8.01 min. ESI-MS: calculated exact mass 667.4; found *m*/*z* 668.4 $[M+H]^+$.

Methyl pivaloyl-L-tyrosylglycylglycyl-L-phenylalanyl-L-leucinate (KK-102)

Pivaloyl-L-tyrosine **8** (0.2 g, 0.75 mmol) was reacted with methyl glycylglycyl-L-phenylalanyl-L-leucinate **7** (0.3 g, 0.75 mmol) according to procedure A to yield compound the crude residue and was purified by flash chromatography eluting with methanol/DCM (1:9, *v*/*v*) to yield KK-102 as a white solid (0.38 g, 77.5%). ^{1}H-NMR (400 MHz, 8:2 DMSO-d_6:H_2O) δ 7.22-7.16 (m, 5H), 6.94 (d, *J* = 6.4 Hz, 2H), 6.90 (d, *J* = 8.1 Hz 2H), 4.48-4.45 (m, 1H), 4.38 (q, *J* = 4.6, 9.5 Hz, 1H), 4.23-4.20 (m, 1H), 3.75-3.61 (m, 4H), 3.55 (s, 3H), 3.01-2.94 (2H, m), 2.77-2.71 (2H, m), 1.54-1.45 (m, 3H), 0.94 (s, 9H), .088 (dd, *J* = 5.7, 22.9 Hz, 6H). ^{13}C-NMR (200 MHz, 8:2 DMSO-d_6:H_2O) δ 178.9, 173.3, 172.8, 171.8, 170.0, 169.2, 155.7, 137.5, 130.6, 129.5, 128.2, 126.9,115.2, 54.7, 54.2, 50.8, 42.5, 42.1, 37.6, 36.3, 27.3, 24.5, 22.9, 21.9. UPLC: retention time = 7.69 min. ESI-MS: calculated exact mass 653.3; found *m*/*z* 654.8 $[M+H]^+$.

Methyl pent-4-enoyl-L-tyrosylglycylglycyl-L-phenylalanyl-L-leucinate (KK-105)

Pent-4-enoyl-L-tyrosine **13** (0.3 g, 1.13 mmol) was reacted with methyl glycylglycyl-L-phenylalanyl-L-leucinate **7** (0.46 g, 1.13 mmol) according to procedure A to yield the crude residue and was purified by flash chromatography eluting with methanol/DCM (1:9, *v*/*v*) to yield KK-102 as a colorless solid (0.45 g, 61.6%). ^{1}H-NMR (400 MHz, 8:2 DMSO-d_6:H_2O) δ 7.23-7.16 (m, 5H), 6.99 (d, *J* = 8.1 Hz, 2H), 6.61, (d, *J* = 7.9 Hz, 2H), 5.64-5.54 (m, 1H), 4.90-4.82 (m, 2H), 4.49-4.46 (m, 1H), 4.36-4.34 (m, 1H), 4.23-4.20 (m, 1H), 3.67-3.60 (m, 4H), 3.56 (s, 3H), 3.02-2.80 (m, 2H), 2.78-2.72 (m, 1H), 2.65-2.55 (m, 1H), 2.13-2.08 (m, 4H), 1.56-1.44 (m, 3H), 0.81 (dd, *J* = 5.8, 22.88, Hz, 6H); ^{13}C-NMR (200 MHz, 8:2 DMSO-d_6:H_2O) δ 173.4, 173.3, 173.2, 172.6, 171.6, 169.6, 168.9, 155.9, 137.8, 130.4, 129.6, 128.5, 126.8, 115.2, 115.5, 54.7, 54.0, 52.3, 50.8, 42.4, 42.1, 37.8, 36.8, 35.6, 31.5, 28.8, 25.5, 24.6, 23.0, 22.4, 21.7, 14.3. UPLC: retention time = 8.72 min. ESI-MS: calculated exact mass 651.3; found *m*/*z* 652.8 [M+H] and 673.8 $[M+Na]^+$.

Methyl octanoyl-L-tyrosylglycylglycyl-L-phenylalanyl-L-leucinate (KK-108)

Octanoyl-L-tyrosine **12** (0.25 g, 0.81 mmol) was reacted with methyl glycylglycyl-L-phenylalanyl- L-leucinate **7** (0.32 g, 0.81 mmol) according to procedure A to yield crude residue and was purified by flash chromatography eluting with methanol/DCM (1:9, *v*/*v*) to yield KK-102 as a white solid (0.35 g, 64.8%). ^{1}H-NMR (400 MHz, 8:2 DMSO-d_6:H_2O) δ 7.25-7.17 (m, 5H), 6.99 (d, *J* = 8.2 Hz, 2H), 6.61, (d, *J* = 8.0 Hz, 2H), 4.48 (q, *J* = 4.5, 9.5 Hz, 1H), 4.35 (q, *J* = 4.5, 9.7 Hz, 1H) 4.24-4.20 (m, 1H), 3.69-3.60 (m, 4H), 3.56 (s, 3H), 3.03-2.90 (m, 2H), 2.79-2.73 (m, 1H), 2.66-2.60 (m, 1H), 2.02 (t, *J* = 7.33 Hz), 1.57-1.45 (m, 3H), 1.32-1.29 (m, 2H), 1.18-1.11 (m, 6H), 1.01 (m, 2H), 0.85-0.78 (m, 9H); ^{13}C-NMR (200 MHz, 8:2 DMSO-d_6:H_2O) δ 173.5, 173.4, 173.1, 172.1, 170.2, 169.5, 156.0, 137.9, 130.7, 129.8, 128.8, 128.5, 127.1, 115.8, 115.5, 55.3, 54.4, 52.6, 51.2, 42.9, 42.5, 38.0, 36.9, 34.9, 29.6, 24.8, 23.2, 21.9, 14.1. UPLC: retention time = 7.57 min. ESI-MS: calculated exact mass 695.4; found *m*/*z* 696.4 $[M+H]^+$.

Methyl (cyclohexanecarbonyl)-L-tyrosylglycylglycyl-L-phenylalanyl-L-leucinate (KK-112)

Cyclohexanecarbonyl)-L-tyrosine **14** (0.3 g, 1.0 mmol) was reacted with methyl glycylglycyl-L-phenylalanyl-L-leucinate **7** (0.41 g, 1.0 mmol) according to procedure A to yield the crude residue and was purified by flash chromatography eluting with methanol/DCM (1:9, *v*/*v*) to yield KK-102 as a white solid (2.5 g, 76.0%). ^{1}H-NMR (400 MHz, 8:2 DMSO-d_6:H_2O) δ 7.21-7.17 (m, 5H), 6.98 (d, *J* = 7.9 Hz, 2H), 6.62 (d, *J* = 8.0 Hz, 2H), 4.47 (q, *J* = 4.3, 9.1, 1H), 4.37 (q, *J* = 4.5, 9.5, 1H), 4.24-4.21 (m, 1H) 3.73-3.61 (m, 4H), 3.57 (s, 3H), 3.02-2.90 (m, 2H), 2.79-2.62 (m, 2H) 2.08 (m, 1H), 1.55-1.42 (m, 8H), 1.19-1.08 (m, 5H), 0.82 (dd, *J* = 5.6, 22.7, 6H); ^{13}C-NMR (200 MHz, 8:2 DMSO-d_6:H_2O) δ 175.9, 173.4, 172.7, 171.9, 169.7, 169.0, 156.3, 138.4, 130.7, 129.9, 128.8, 128.7, 126.9, 115.4, 54.7, 53.3, 52.5, 51.1, 44.3, 42.8, 42.5, 38.3, 37.2, 29.9, 29.4, 26.1, 26.0, 25.8, 24.9, 23.4, 22.0. UPLC: retention time = 8.01 min. ESI-MS: calculated exact mass 679.4; found *m*/*z* 680.4 $[M+H]^+$.

Pivaloyl-L-tyrosylglycylglycyl-L-phenylalanyl-L-leucine (KK-103)

To a solution of KK-102 (0.35 g, 0.58 mmol) in THF (10 mL) a solution of LiOH (0.21 g, 3.48 mmol) in H_2O (5 mL) added dropwise according to procedure C. The resultant crude residue was purified by flash chromatography using methanol/DCM (1:9, *v*/*v*) to yield KK-103 as a white solid (0.25 g, 65.7%). Optical rotation: $[\alpha]_D{}^{20}$ = -17.8° (*c* 1.0, CH_3OH); ^{1}H-NMR (400 MHz, 8:2 DMSO-d_6:H_2O) δ 7.24-7.15 (m, 5H), 6.96 (d, *J* = 8.2 Hz, 2H), 6.60 (d, *J* = 8.0 Hz, 2H), 4.47 (q, *J* = 4.1, 9.5 Hz, 1H), 4.37 (q, *J* = 4.3, 9.2 Hz, 1H), 4.18-4.14 (m, 1H), 3.71-3.50 (m, 4H), 3.02-2.93 (m, 2H), 2.76-2.71 (m, 2H), 1.56-1.46 (m, 3H), 0.93 (m, 9H), 0.81 (dd, *J* = 5.9, 23.9 Hz, 6H). ^{13}C-NMR (200 MHz, 8:2 DMSO-d_6:H_2O) δ 179.2, 174.7, 173.2, 173.1, 172.0, 170.3, 169.6, 156.0, 138.0, 130.9, 129.8, 128.9, 128.5, 127.2, 115.5, 55.0, 54.4, 51.1, 42.8, 42.4, 38.0, 36.6, 27.6, 24.9, 23.4, 21.9. UPLC: retention time = 5.42 min. ESI-MS: calculated exact mass 639.3; found *m*/*z* 640.4 $[M+H]^+$ and 662.1 $[M+Na]^+$. UPLC purity: >95%.

2.4. Binding Poses and Energetics of Leu-ENK and KK103 Suggested by Molecular Dynamics (MD) Simulations Following Molecular Docking

The ligands Leu-ENK and KK-103 were docked individually into the binding site of both active (PDBID: 6PT2) and inactive (PDBID: 4EJ4) DOR structures by AutoDock Vina software (Molecular Graphics Lab, La Jolla, CA, USA).[16] using an exhaustiveness of 200. The pose with the lowest energy (i.e. the highest affinity) obtained from the docking results were selected for creating a water box of 62.3 × 62.3 × 96.0 Å.[3] It contains TIP3P (transferable intermolecular potential with 3 points) explicit solvent molecules (i.e. water), neutralizing ions and a liganded DOR that is embedded in the POPC (1-palmitoyl-2-oleoyl-sn-glycero-3-phosphoserine) lipid membrane, facilitated by CHARMM-GUI Bilayer Membrane Builder software (Lehigh Universiy, Bethlehem, PA).[17] OpenMM software[18] (SimTK, National Institute of Health, MD) running with CHARMM36 forcefield[19] was used for all simulations at a temperature of 25.15 °C and a pressure of 1 bar, where the ligand forcefield was generated by CGenFF.[20] X-Y

isotropic Monte-Carlo barostat was set for the membrane system. The restrained simulations were first performed to relax the environment and prevent the distortion of the ligand-DOR complex. Production runs without any restraints were carried out for at least 100 ns to equilibrate the system. Snapshots of the DOR-ligand complex were taken from the last 20 ns of the ε100 ns equilibrated trajectories for binding free energy evaluation using the MM/GBSA method7, implemented in the MMPBSA.py script of AmberTool20 (AMBER Software, San Francisco, CA, USA).[21] For each ligand, the binding free energies with active and inactive DOR were compared to predict the ligand type (agonist/antagonist); for the same ligand type, their GBSA (generalized Born surface-area solvation) energies were used to infer binding affinity (the lower, the stronger). The MM/GBSA (molecular mechanics with generalized Born surface-area solvation) energy can further be broken down to infer peptide-DOR interaction at the residue level, pointing out which residues in DOR contribute the most binding affinity to interact with Leu-ENK or KK-103.

References

1. Shakespeare, W.; Yang, M.; Bohacek, R.; Cerasoli, F.; Stebbins, K.; Sundaramoorthi, R.; Azimioara, M.; Vu, C.; Pradeepan, S.; Metcalf, C.; et al. Structure-based design of an osteoclast-selective, nonpeptide Src homology 2 inhibitor with in vivo antiresorptive activity. *Proc. Natl. Acad. Sci. U. S. A.* **2000**, *97*, 9373–9378, doi:10.1073/pnas.97.17.9373.
2. Shechter, Y.; Heldman, E.; Sasson, K.; Bachar, T.; Popov, M.; Fridkin, M. Delivery of neuropeptides from the periphery to the brain: Studies with enkephalin. *ACS Chem. Neurosci.* **2010**, *1*, 399–406, doi:10.1021/cn100001j.
3. Mosberg, H.I.; Hurst, R.; Hruby, V.J.; K, G.; Yamamura, H.I.; Galligan, J.J.; Burks, T.F. Bis-penicillamine enkephalins possess highly improved specificity toward δ opioid receptors. *Proc. Natl. Acad. Sci. U. S. A.* **1983**, *80*, 5871–5874, doi:10.1073/pnas.80.19.5871.
4. Harris, H.M.; Eans, S.O.; Ganno, M.L.; Davis, J.C.; Dooley, C.T.; McLaughlin, J.P.; Nefzi, A. Antinociceptive activity of thiazole-containing cyclized DAMGO and Leu-(Met) enkephalin analogs. *Org. Biomol. Chem.* **2019**, *17*, 5305–5315, doi:10.1039/c9ob00882a.
5. Beaudeau, J.L.; Blais, V.; Holleran, B.J.; Bergeron, A.; Pineyro, G.; Guérin, B.; Gendron, L.; Dory, Y.L. N-Guanidyl and C-Tetrazole Leu-Enkephalin Derivatives: Efficient Mu and Delta Opioid Receptor Agonists with Improved Pharmacological Properties. *ACS Chem. Neurosci.* **2019**, *10*, 1615–1626, doi:10.1021/acschemneuro.8b00550.
6. Mollica, A.; Pinnen, F.; Stefanucci, A.; Feliciani, F.; Campestre, C.; Mannina, L.; Sobolev, A.P.; Lucente, G.; Davis, P.; Lai, J.; et al. The cis-4-amino-l-proline residue as a scaffold for the synthesis of cyclic and linear endomorphin-2 analogues. *J. Med. Chem.* **2012**, *55*, 3027–3035, doi:10.1021/jm201402v.
7. Liau, W.T.; Kasko, A.M. Poly(methyl 6-acryloyl-β- d -glucosaminoside) as a Cationic Glycomimetic of Chitosan. *Biomacromolecules* **2017**, *18*, 4133–4140, doi:10.1021/acs.biomac.7b01191.
8. Castillo Gomez, J.D.; Hagenbach, A.; Abram, U. Propargyl-Substituted Thiocarbamoylbenzamidines of Technetium and Rhenium: Steps towards Bioconjugation with Use of Click Chemistry. *Eur. J. Inorg. Chem.* **2016**, *2016*, 5427–5434, doi:10.1002/ejic.201601039.
9. Martyn, D.C.; Vernall, A.J.; Clark, B.M.; Abell, A.D. Ring-deactivated hydroxyalkylpyrrole-based inhibitors of α-chymotrypsin: Synthesis and mechanism of action. *Org. Biomol. Chem.* **2003**, *1*, 2103–2110, doi:10.1039/b302411c.
10. Singh, P.; Kaur, S.; Kaur, J.; Singh, G.; Bhatti, R. Rational Design of Small Peptides for Optimal Inhibition of Cyclooxygenase-2: Development of a Highly Effective Anti-Inflammatory Agent. *J. Med. Chem.* **2016**, *59*, 3920–3934, doi:10.1021/acs.jmedchem.6b00134.
11. Zhang, C.; Liu, S.S.; Sun, B.; Tian, J. Practical Peptide Synthesis Mediated by a Recyclable Hypervalent Iodine Reagent and Tris(4-methoxyphenyl)phosphine. *Org. Lett.* **2015**, *17*, 4106–4109, doi:10.1021/acs.orglett.5b02045.
12. Bilcer, G.M.; Kelly, T.A. Granzyme b directed imaging and therapy 2019.
13. Georgiades, S.N.; Clardy, J. Synthetic libraries of tyrosine-derived bacterial metabolites. *Bioorganic Med. Chem. Lett.* **2008**, *18*, 3117–3121, doi:10.1016/j.bmcl.2007.10.058.
14. Phoon, C.W.; Somanadhan, B.; Heng, S.C.H.; Ngo, A.; Ng, S.B.; Butler, M.S.; Buss, A.D.; Sim, M.M. Isolation and total synthesis of gymnastatin N, a POLO-like kinase 1 active constituent from the fungus Arachniotus punctatus. *Tetrahedron* **2004**, *60*, 11619–11628, doi:10.1016/j.tet.2004.09.046.
15. Santiago, N.B.; Haas, S.; Leone-Bay, A.; Milstein, S.J.; Barantsevitch, E. Compositions for the delivery of antigens 1998.
16. D.A. Case; K. Belfon; I.Y. Ben-Shalom; S.R. Brozell; D.S. Cerutti; T.E. Cheatham; III; V.W.D. Cruzeiro; T.A. Darden; R.E. Duke; et al. *Amber 2020*; 2020;
17. Jo, S.; Lim, J.B.; Klauda, J.B.; Im, W. CHARMM-GUI membrane builder for mixed bilayers and its application to yeast membranes. *Biophys. J.* **2009**, *97*, 50–58, doi:10.1016/j.bpj.2009.04.013.
18. Eastman, P.; Swails, J.; Chodera, J.D.; McGibbon, R.T.; Zhao, Y.; Beauchamp, K.A.; Wang, L.P.; Simmonett, A.C.; Harrigan, M.P.; Stern, C.D.; et al. OpenMM 7: Rapid development of high performance algorithms for molecular dynamics. *PLoS Comput. Biol.* **2017**, *13*, doi:10.1371/journal.pcbi.1005659.

19. Huang, J.; Mackerell, A.D. CHARMM36 all-atom additive protein force field: Validation based on comparison to NMR data. *J. Comput. Chem.* **2013**, *34*, 2135–2145, doi:10.1002/jcc.23354.
20. Vanommeslaeghe, K.; Hatcher, E.; Acharya, C.; Kundu, S.; Zhong, S.; Shim, J.; Darian, E.; Guvench, O.; Lopes, P.; Vorobyov, I.; et al. CHARMM general force field: A force field for drug-like molecules compatible with the CHARMM all-atom additive biological force fields. *J. Comput. Chem.* **2010**, *31*, 671–690, doi:10.1002/jcc.21367.
21. Miller, B.R.; McGee, T.D.; Swails, J.M.; Homeyer, N.; Gohlke, H.; Roitberg, A.E. MMPBSA.py: An efficient program for end-state free energy calculations. *J. Chem. Theory Comput.* **2012**, *8*, 3314–3321, doi:10.1021/ct300418h.
